# The incidence and epidemiology of patellar fractures following medial patellofemoral ligament reconstruction: a systematic review and meta-analysis

**DOI:** 10.1007/s00402-026-06440-y

**Published:** 2026-08-03

**Authors:** Diego Agustín Abelleyra Lastoria, Ashton Hunt, Carlo Keenan Ross, Meri Yemane, Sophie Keynes, Aishwarya Ghosh, Caroline Blanca Hing

**Affiliations:** City St Georges, University of London, London, United Kingdom

**Keywords:** Patellar instability, Medial patellofemoral ligament, Reconstruction, Fracture

## Abstract

**Introduction:**

Patellar instability can arise from a traumatic event with anatomical predisposing factors increasing the risk of dislocation. Medial patellofemoral ligament reconstruction (MPFLR) is commonly performed to treat patellar instability. The aim of this systematic review was to determine the incidence of and risk factors for patellar fractures after MPFLR.

**Materials and methods:**

The protocol for this review was registered on PROSPERO with registration number CRD420251066107. The PRISMA 2020 checklist was followed. Electronic databases and conference proceedings were searched to the 15th of May 2025. Studies were eligible if they reported incidence of patellar fractures after MPFLR. A random-effects meta-analysis was performed. Included studies were appraised using tools respective of study design.

**Results:**

A total of 4,972 records were screened in the initial search. A total of 110 studies satisfied the inclusion criteria, evaluating 115,496 patients. The evidence was moderate to low in quality. The incidence of patellar fracture ranged from 0 to 9.50%, with a pooled incidence of 1.17% (95% confidence interval [CI] 0.76- 1.68%). There was no difference in incidence of patellar fractures between patients who did and did not undergo bone tunnel fixation (0.94%, 95% CI 0.69–1.24 vs. 1.00%, 95% CI 0.15–2.44%, respectively). There was no difference in fracture incidence between patients who underwent concomitant procedures (1.26%, 95% CI 0.51–2.32) and those who underwent MPFLR alone (0.53%, 95% CI 0.13–1.16%).

**Conclusion:**

Patellar fractures are a known complication of MPFLR, with a higher incidence observed in patients undergoing this procedure compared to the baseline population risk. Current evidence suggests its incidence is not affected by tunnel location or the performance of concomitant procedures, though further prospective studies are required to corroborate this. Appropriate post-operative follow-up is required to monitor the occurrence of patellar fractures during the post-operative period.

## Introduction

Patellar instability can lead to patellar dislocation, which accounts for 2–3% of injuries of the knee joint [1]. It has an incidence of 6 in every 100,000 patients per year among adults [2]. Recurrent dislocations occur in 15–44% of patients [3]. Patellar dislocation can result in femoral condyle contusion and rupture of the medial patellofemoral ligament (MPFL), with associated tearing of the medial retinaculum [1, 4].

The MPFL inserts onto the upper medial aspect of the patella [5]. The MPFL helps prevent lateral dislocation or subluxation [6]. This may be damaged when turning or twisting the leg, predisposing to lateral patellar dislocation [5]. Injury to the MPFL after patellar dislocation is very common, with an incidence over 90% [7]. Though this may heal with immobilization, the ligament can become lengthened and loosened, leading to lateral patellar instability and increased risk of dislocation [8].

Medial patellofemoral ligament reconstruction is a common surgical procedure for treating patellar instability [9]. This involves replacing the damaged ligament with a tendon autograft, allograft or synthetic graft [9]. This is fixed to bone by either inlay or onlay techniques, with the former anchoring the graft into the tunnel, and the latter with the graft not anchored in a tunnel [10]. This can be performed in isolation, or in combination with bony procedures such as tibial tubercle osteotomy, de-rotational femoral osteotomy, or trochleoplasty [8].

A postulated complication of MPFLR is patellar fracture, with the use of bone tunnels or anchors suggested as a pathophysiological mechanism [11]. Patellar fractures can lead to significant long-term symptoms and limitations, including stiffness, extension weakness, and patellofemoral arthritis [12]. A recent systematic review estimated the incidence of patellar fracture following MPFLR to be between 0 and 8.3%. However, no statistical methods were employed to determine its incidence or risk factors [13]. Awareness of these parameters could allow clinicians to hold informed discussions with patients and relatives regarding consequences of operative intervention. Therefore, the aim of this systematic review was to determine the incidence and risk factors of patellar fractures after MPFLR.

## Methods

The protocol for this review was registered on PROSPERO with registration number CRD420251066107. The PRISMA 2020 checklist was followed [14].

### Study eligibility

Study eligibility was determined by following pre-specified criteria. Studies were eligible if they reported on the incidence of or risk factors for patellar fractures after MPFLR. Both full-texts and abstracts were included. Studies not clearly reporting whether patellar fracture occurred following MPFLR were excluded. Eligible study designs comprised case series, case-control, cross-sectional, and cohort studies, as well as randomised controlled trials.

Both retrospective and prospective studies were eligible. Cadaveric studies and scoping or systematic reviews were excluded, along with letters to the editor, case reports, computational models, and animal studies. There were no constraints based on publication status, language or patient demographics. Eligibility assessment was performed by two reviewers (DAAL, AH).

### Search strategy and data extraction

Embase, Global Health and MEDLINE were searched via OVID. Electronic databases were searched from their inception to the 15th of May 2025. A grey literature search of currently registered studies and conference proceedings was conducted to identify titles of potentially relevant full-text studies. Currently registered studies were reviewed using the following databases: the NIHR Portfolio, the ISRCTN registry, the UK National Research Register Archive, OpenSIGLE, and the World Health Organization International Clinical Trials Registry Platform. Conference proceedings from the European Federation of National Associations of Orthopaedics and Traumatology, the British Trauma Society, and British Orthopaedic Association were searched. Searches were conducted twice for quality assurance. The search strategy is presented in Appendix 1.

### Methodological appraisal

Level of evidence and risk of bias of included studies were evaluated independently by five reviewers (AH, CKR, MY, SK, AG), with a separate reviewer resolving any disagreements (DAAL). The level of evidence of the studies presented was determined using the March 2009 Oxford Centre for Evidence-Based Medicine: Levels of Evidence [15]. The Institute of Health Economics case series studies quality appraisal checklist was used to determine risk of bias for case series [16]. In addition, the CLARITY tools for cohort and case-control studies were used [17], as well as the Cochrane collaboration tools for randomised controlled trials and non-randomised studies of intervention [18, 19]. We used funnel plots to visually assess the presence of small study bias for analyses pooling three or more studies.

### Data analysis

Baseline characteristics including number of patients, number of knees, patient sex, age, follow-up duration, and comorbidities were extracted (Table 1). Where sufficient (at least two) and homogeneous studies reported on the same outcome, a random-effects meta-analysis was performed using MetaXL version 5.3 software (EpiGear International Pty Ltd, Wilston, Queensland, Australia). Analyses were conducted at the patient level given that many studies did not report the number of knees included.

**Table 1:** Twenty-one studies did not report the number of knees included. Ninety-four studies reported patient sex (38,134 males, 33.4%, and 76,073 females, 66.6%). Patient age ranged from six to 83 years. The geographical distribution in the studies was included, with the United States (24 studies), China (23 studies), and the United Kingdom (10 studies) being the most represented countries. Study baseline characteristics

Study	Study location	Number of patients	Number of knees	Males	Females	Paediatric population only?	Mean age (years)	Age standard deviation (years)	Median age (years)	Age range (years)	Comorbidities	Follow-up
Acharya et al. [46]	Kathmandu, Nepal	18	18	11	7	NR	NR	NR	NR	NR	NR	NR
Allahabadi and Pandya [101]	San Francisco, United States	20	24	6	14	NR	15.7	2.1	NR	11.5 to 19.6	NR	2 years
Ambrožič and Novak [72]	Ljubljana, Slovenia	29	31	15	14	No	26.2	6.4	NR	17.5 to 40.6	NR	6.4 ± 1.2 years
Arshi et al. [23]	United States	6190	NR	2501	3689	No	NR	NR	NR	10 to 34	NR	1 year
Astur et al. [24]	Sao Paulo, Brazil	58	NR	30	28	No	29.5	NR	NR	18 to 45	None	5 years
Bachman et al. [52]	United States	31	37	NR	NR	Yes	12.4	NR	NR	NR	Genu valgum	27.7 ± 21.0 months
Chatterton et al. [83]	Denmark	23	23	6	17	NR	23.0	8.6	NR	NR	NR	44 months
Christiansen et al. [93]	Århus, Denmark	44	44	15	29	No	NR	NR	22	12 to 47	High-grade patellar/trochlear cartilage lesions in some patients	12 to 32 months
Deie et al. [102]	Hiroshima, Japan	29	31	5	24	No	22.2	NR	NR	12 to 34	None	2 to 5 years
Deo et al. [103]	Great Yarmouth, UK	85	85	27	58	No	28.1	9.4	25	17 to 55	None	1 to 9 years (mean 4.84 years)
Devgan et al. [104]	Haryana, India	20	20	8	12	No	21.0	NR	NR	17 to 34	NR	26.4 months (range 23–30 months)
Dong et al. [105]	Hebei, China	45	45	16	29	No	22.7	3.3	NR	17 to 29	All patients had patella alta	Mean 24 months (range 22–25 months)
Enderlein et al. [106]	Aarhus, Denmark	224	240	78	162	No	NR	NR	23	14 to 46	33% of patients had previous procedures performed on the affected knee (including arthroscopy, distal realignment, lateral release and MPFL reinsertion)	1 year
Ercan et al. [26]	Ankara, Turkey	80	NR	20	20	No	NR		15	10 to 28	None	Median 46.5 months Rane (24–74 months)
Fadel and Hosni [71]	Egypt	34	34	5	29	No	19.4	2.7	NR	NR	None	Mean 28.7 months
Fisher et al. [73]	Ohio, United States	34	36	22	14	Yes	16.3	1.8	NR	11 to 19	NR	Mean 35.9 ± 15.2 months
Fitzgerald et al. [27]	United States	29,539	NR	9,589	19,950	No	NR	NR	NR	14 to 40	NR	NR
Fuji et al. [107]	Kyoto, Japan	24	27	6	18	No	23.9	9.4	NR	11 to 46	Trochlear dysplasia in 24 knees	1 year
Gomes [88]	Rio Grande do Sul, Brazil	30	30	12	18	No	29.0	NR	NR	17 to 50	Patellofemoral arthritis (*n* = 2), chondromalacia (*n* = 14), meniscal lesion (*n* = 1), chondral lesion (*n* = 1)	Mean 39 months
Gorbaty et al. [29]	North Carolina, United States	486	NR	170	316	No	NR	NR	20	16 to 29	NR	Mean 4.2 years (range 1–10 years)
Goyal et al.[62]	Telangana, India	23	23	11	12	No	NR	NR	NR	20 to 55	None	Mean 31.5 months (range 2–6 years)
Han et al. [109]	Lanzhou, China	52	59	19	33	No	24.3	NR	NR	15 to 41	NR	Mean 5.7 years
Han et al. [30]	Xinjiang, China	57	NR	19	38	No	NR	NR	NR	16 to 26	NR	NR
Hiemstra et al. [110]	Cleveland, Ohio, United States	363	461	NR	NR	NR	NR	NR	NR	NR	NR	2 years
Hohn and Pandya [53]	California, United States	25	25	7	18	Yes	16.0	2	NR	NR	None	Mean 24 months (range 12–44 months)
Hong et al. [77]	Hwaseong, Republic of Korea	27	27	16	11	No	22.0	6.4	NR	14 to 32	NR	5 years
Hopper et al. [94]	Glasgow, United Kingdom	68	72	18	50	No	23.9	NR	NR	14 to46	NR	Mean 31.3 months (range 13–72 months)
Howells et al. [89]	Bristol, United Kingdom	193	211	84	109	No	26.0	NR	NR	16 to49	10 patients (4.7%) with chronic low-back pain (3), depression (3), complex regional pain syndrome (3) or fibromyalgia (1)	Mean 16 months (range 6–42 months)
Howells and Eldridge [51]	Bristol, United Kingdom	75	75	7	68	No	25.0	NR	NR	17 to49	8/25 hypermobile patients had joint hypermobility syndrome; otherwise None reported	Mean 15 months (range 6–30 months)
Kang et al. [31]	Hebei, China	45	NR	18	27	No	26.6	5.8	NR	NR	None	Mean 33.7 months
Kim et al. [32]	Hwasun, Sotuh Korea	81	NR	42	39	No	22.1	NR	NR	NR	None	Mean 25.2 months (range12–53 months)
Kita et al. [91]	Osaka, Japan	24	25	6	18	No	22.7	NR	NR	13 to 43	NR	1 year
Kita et al. [90]	Japan	42	44	9	33	No	25.4	NR	NR	13 to 43	NR	24 months
Kita et al. [113]	Sakai, Japan	23	23	6	17	No	24.1	9.4	NR	14 to 53	NR	Mean 3.2 years (range 2–9 years)
Klueh et al. [54]	Michigan, United States	48	64	26	22	Yes	14.0	1.8	NR	9 to 17	NR	1 year
Kodkani et al. [67]	Mumbai, Maharashtra, India	50	56	15	39	No	20.6	NR	NR	9 to 48	*Type C trochlear dysplasia in 12 knees*,* 31 had type A/B. Five knees had prior failed stabilization surgeries*	Mean 26 months
Kumahashi et al. [55]	Izumo, Japan	5	5	2	3	Yes	13.6	NR	14	11 to 15	NR	Mean 22 months (12–71 months)
Kumahashi et al. [114]	Izumo, Japan	17	19	3	14	No	20.0	NR	NR	14 to 38	1 patient with generalised joint laxity	Mean 27.8 months (range 24 − 36 months
Kumar et al. [9]	Tamil Nadu, India	30	30	13	17	No	NR	NR	18	11 to 50	NR	Mean 25 months (range 14–38 months)
Kumar et al. [56]	San Diego, United States	59	59	21	38	Yes	15.2	1.7	NR	NR	NR	Mean 4.1 ± 1.9 years
Lin et al. [115]	Kaohsiung, Taiwan	18	18	NR	NR	NR	NR	NR	NR	NR	NR	Mean 35 months
Liu et al. [79]	New York, United States	121	121	33	88	No	23.8	NR	NR	12 to 57	NR	Mean 44 months
Maione et al. [116]	Milan, Italy	46	51	17	46	No	24.1	7.4	NR	16 to 46	None	Mean 8.9 ± 2.1 years
Marigi et al. [117]	Minnesota, United States	56	62	NR	NR	No	17.0	NR	NR	14 to 22	NR	Mean 6.6 years (range, 2.0–18.8 years)
Markes et al. [33]	United States	70,070	NR	22,884	47,186	No	NR	NR	NR	NR	NR	2 years
Matthews and Schranz [118]	Exeter, UK.	21	25	12	9	No	NR	NR	24	17 to 44	None	Mean 30 months
Mikashima et al. [44]	Japan	24	24	10	14	No	21.8	4.9	NR	13 to 24	None	2 years
Mochizuki et al. [82]	Tokyo, Japan	24	24	6	18	No	25.4	9.3	NR	NR	None	2 years
Mohammed et al. [119]	United Kingdom	53	58	20	33	No	NR	NR	NR	NR	NR	6 months
Monllau et al. [120]	Barcelona, Spain	35	36	17	19	No	25.6	9.4	NR	NR	NR	27 months
Moran et al. [85]	Charlottesville, United States	560	635	NR	NR	No	NR	NR	NR	NR	NR	2 years
Mulliez et al. [68]	Ghent & Antwerp, Belgium	124	129	48	81	No	22.8	7.7	NR	11 to 43	None	Mean 34.5 ± 15.3 months
Nazeer et al. [69]	United Kingdom & India	29	29	7	22	No	30.4	NR	NR	17 to 49	None	30 months
Neyret et al. [45]	France	32	35	NR	NR	NR	NR	NR	NR	NR	NR	NR
Niu et al. [122]	Shijiazhuang, China	64	64	17	47	No	NR	NR	NR	NR	None	4 years
Niu et al. [121]	Hebei, China	30	30	10	20	No	25.0	6.9	NR	16 to 35	None	25 months
Ntagiopoulos et al. [34]	Athens, Greece	24	NR	8	16	Yes	13.0	2.29	NR	9 to 16	NR	Mean 38.66 months (range 24–86 months)
Özel et al. [123]	Turkey	51	51	23	28	No	23.0	NR	NR	NR	NR	2 years
Palacios et al. [35]	Buenos Aires, Argentina	19	NR	9	10	No	25.0	NR	NR	NR	None	3 years
Panni et al. [92]	Italy & United Kingdom	48	51	11	37	No	28.0	NR	NR	16 to 60	Chondral lesions present in all knees	Mean 33 months
Parikh et al. [95]	Cincinnati, United States	154	179	63	91	Yes	14.5	2.6	NR	6 to 21	None	Mean 16.2 months
Park et al. [36]	Yale University, United States	73	NR	18	55	NR	21.5	NR	NR	NR	NR	3 months
Perkins et al. [37]	Georgia, United States	82	NR	34	48	Yes	15.3	NR	NR	12 to 18	NR	Mean 31 months
Reddy et al. [57]	United Kingdom	57	76	19	57	Yes	14.0	NR	NR	7to16	3 patients had Down’s syndrome, 2 had Ehler–Danlos syndrome, 2 had cerebral palsy, 1 Jacobsen syndrome, and 2 had severe learning difficulties.	Mean 3 years (range 1–4 years)
Rhatomy et al. [38]	Klaten, Indonesia	8	NR	7	1	No	30.0		19	17 to 23	NR	2 years
Ronga et al. [63]	London, United Kingdom	28	28	21	7	No	32.5	11.4	NR	19 to 40	Osteochondral lesions (< 15 mm) in 11/28 patients, Grade III–IV patellofemoral degenerative change in 10/28.	Mean 3.1 years (range, 2.5–4 years)
Sanguanjit et al. [39]	Pathum Thani, Thailand	43	NR	19	24	No	NR	NR	NR	12 to 58	None	Mean 46.9 months (range 24–77 months)
Shankar et al. [40]	New York, United States	10	NR	1	9	No	29.1	NR	NR	NR	NR	Mean 28.3 months
Sherman et al. 47]	Missouri, United States	87	95	25	62	No	NR	NR	NR	18 to 55	NR	6 months
Sherman et al. [49]	Missouri, United States	25	29	NR	NR	Yes	15.6	NR	NR	10.5 to 17.8	NR	NR
Sim et al. [125]	South Korea	12	11	5	6	No	18.6	4.4	NR	13 to 28	NR	2 years
Song et al. [78]	Hwaseong, South Korea	20	20	10	10	No	NR	NR	21	13 to 34	None	Mean 35.5 (range 25–49 months)
Song et al. [64]	Exeter, United Kingdom	13	13	7	6	No	28.8	NR	NR	25 to 49	None	Median 34.5 months
Steiner et al. [75]	United States & Argentina	34	34	12	22	No	27.0	NR	NR	NR	NR	Mean 66.5 months (range 24–130 months)
Sundararajan et al. [41]	Coimbatore, India	22	NR	5	17	No	29.0	NR	NR	15 to 55	NR	Mean 30 months (17–43 months)
Tang et al. [126]	China	16	16	2	14	No	16.0	NR	NR	11 to 27	NR	Mean 16.4 months (range 10–24 months)
Teküstün et al. [76]	Manisa, Turkey	8	11	2	6	No	19.8	NR	NR	14 to 30	High grade trochlear dysplasia in 1 patient	Mean 11.3 years (range 6–12 years)
Torkaman et al. [42]	Tehran, Iran	15	NR	6	9	No	26.5	5.97	NR	17 to 38	NR	Mean 17.4 months
Toritsuka et al. [127]	Hyogo, Japan	20	20	9	11	No	23.0	8	NR	13 to 38	None	Mean 30 months
Valkering et al. [43]	United Kingdom	31	NR	10	21	NR	23.9	6.9	nr	NR	NR	range 1.5–5.1 years
van Gennip et al. [65]	Groningen, Netherlands	9	9	2	7	No	NR	NR	75	60 to 83	All patients were post TKA or post revision TKA	Meadian 33 months (range 10–48 months)
von Engelhardt et al. [87]	Germany	30	33	12	21	No	24.0	9	NR	NR	None	Mean 29 ± 23 months
Wang et al. [128]	Shijiazhuang, China	26	26	10	16	No	26.3	4.7	NR	NR	NR	3 years
Wu et al. [129]	Beijing, China	68	68	22	46	No	21.0	NR	NR	16 to 32	None	Median 169.02 ± 14.11 months
Xing et al. [130]	Shijiazhuang, China	31	31	13	18	No	NR	NR	18	15 to 53	None	2 years
Yang et al. [70]	Changchun, China	12	12	NR	NR	NR	NR	NR	NR	NR	NR	Mean 16.4 months
Ye et al. [80]	Shenyang, China	65	65	28	37	No	28.5	NR	NR	NR	None	Range 25–60 months
Yoon et al. [132]	Seoul, South Korea	46	46	18	28	No	24.2	NR	NR	NR	NR	2 years
Yoon et al. [131]	South Korea	55	55	22	33	No	21.9	NR	NR	NR	None	2 years
Yoong et al. [48]	Liverpool, Hospital	43	45	NR	NR	No	26.3	NR	NR	14 to 52	NR	NR
Zein et al. [60]	Minia, Egypt	24	24	8	16	Yes	12.4	1.6	NR	9 to 15	None	Mean 47.7 ± 5.8 months (range 39–56 months)
Zein et al. [59]	Egypt	11	12	9	2	Yes	10.7	2.4	NR	5 to 14	Down syndrome in 1 patient	Mean 40 ± 9.6 months (range 28–56 months)
Zhang et al. [133]	China	20	20	4	16	No	19.0	NR	NR	13 to 31	None	2 years
Zhang et al. [134]	Beijing, China	75	115	5	70	NR	21.6	NR	NR	NR	NR	Mean 25.6 months (range 6–34 months)
Zhang et al. [61]	Wenzhou, China	21	21	9	12	Yes	10.7	NR	NR	8 to 13	NR	2 years (range 24–42 months)
Zhang et al. [50]	Chengdu, China	50	50	15	35	No	21.1	NR	NR	NR	None	2 years
Zheng et al. [81]	Shijiazhuang, China	30	30	14	16	No	18.3	NR	NR	15 to 25	None	2 years
Zhou et al. [66]	Hebei, China	32	32	10	22	No	21.3	3.45	NR	19 to 32	NR	Median 18 months (range 12–26 months)
Zhou et al. [135]	Hebei, China	64	64	30	34	No	22.7	NR	NR	NR	Grade A trochlear dysplasia in 33 patients, Grades B/C/D in 31	Mean 28.4 ± 3.6 months
Zhu et al. [136]	Changsha, China	25	25	5	20	No	19.5	3.7	NR	16 to 28	NR	Mean 24 months (range 24–40 months)
Runer at al, [74]	Innsbruck & Vienna, Austria and Munich, Germany	64	64	34	30	No	20	6.3	NR	NR	None	Mean 28.7 ± 7.5 months
Rai et al. [86]	Bihar, India	40	40	17	23	No	24.48	7.31	NR	NR	NR	1 year
Schiphouwer et al. [124]	Nijmegen, Netherlands	179	192	NR	NR	No	NR	NR	19	10 to 57	NR	6 months
Chilelli et al. [25]	Illinois, United States	3152	NR	1268	1884	No	23.3	NR	NR	2 to 99	NR	1 year
Gao et al. [108]	Beijing, China	66	80	17	49	No	21.3	7.8	NR	13 to 50	Trochlear dysplasia	Mean 66.1 ± 5.5 months (range 60–78 months)
Retzky et al. [58]	New York, United States	56	63	28	32	Yes	14.2	1.8	NR	< 18	Osteochondral fracture (*n* = 15)	6 months
Gondolfo et al. [28]	Parana, Brazil	51	NR	22	29	No	30.8	NR	NR	16 to 57	None	2 years
Deasey et al. [84]	Virginia, United States	352	384	132	220	No	23.65	10.59	NR	NR	None	2 years
Hu et al. [111]	Beijing, China	112	112	38	74	No	23	NR	NR	NR	NR	Mean 56.5 months
Huo et al. [112]	Shijiazhuang, China	130	130	51	79	No	28.5	NR	NR	NR	None	3 years

Data on prevalence were presented as weighted prevalence percentage and 95% CI. A sub-group analysis in children was performed. Patients under 18 years of age were included in this group, as per previous work on paediatric populations [20, 21]. In addition, sensitivity analyses excluding studies with over 1000 or less than 30 patients were performed when applicable. Non-statistically significant differences were considered in cases of overlapping 95% CIs.

The incidence of patellar fracture according to graft type used, the drilling of a bone tunnel, tunnel location, and concomitant procedure performed was calculated. These could only be performed in studies which applied the same technique to all patients, or in studies which reported on the specific techniques used in patients who sustained a post-operative fracture. Fracture incidence according to anchor type used could not be calculated given the high variability of techniques between studies and within the patient population of individual studies.

**Fig. 1:**
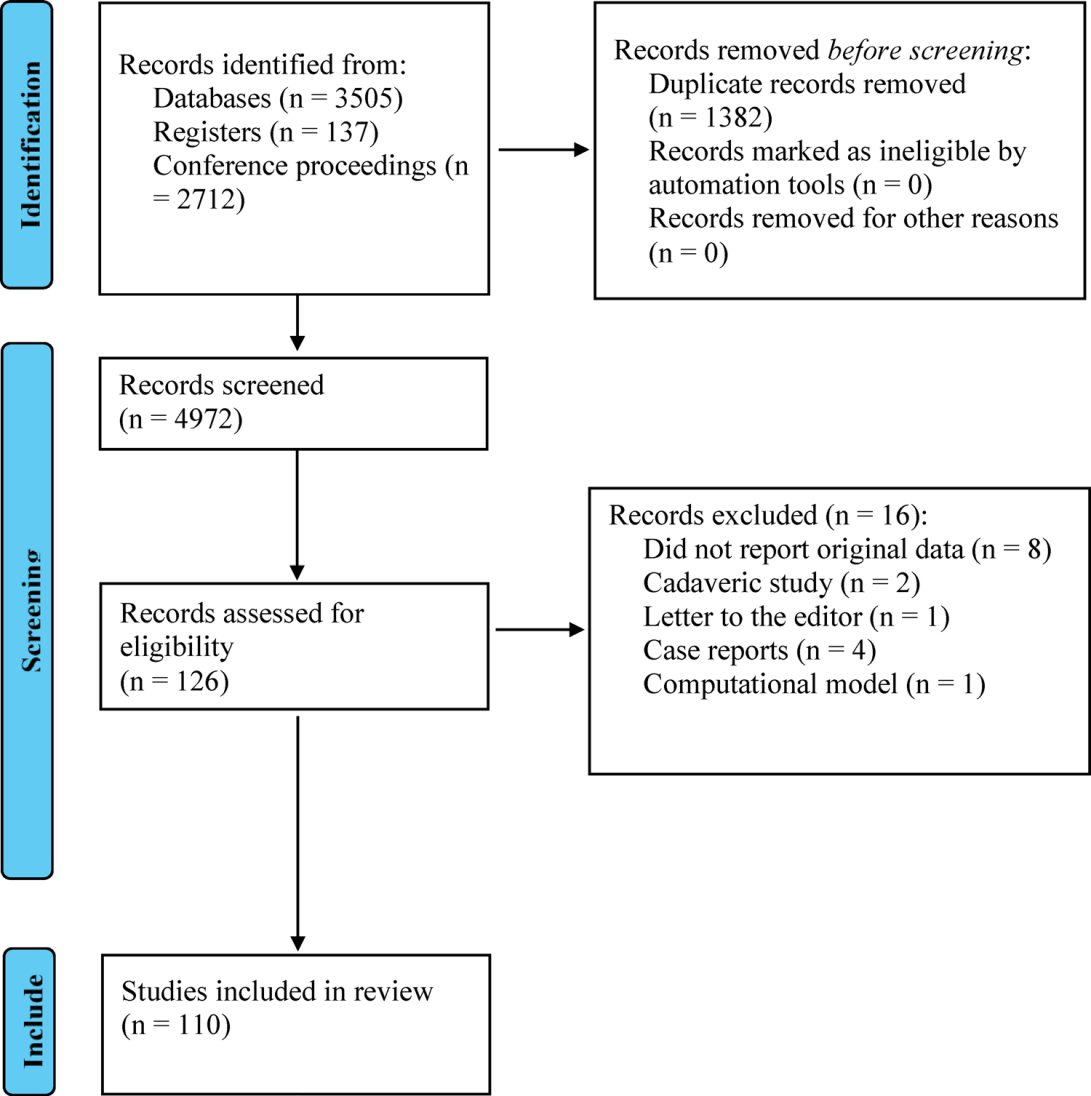
A total of 4972 records were screened, with 126 potentially eligible articles identified. Sixteen were excluded on the basis of the pre-specified exclusion criteria. A total of 110 studies evaluating 115,496 patients were included. PRISMA diagram depicting the study collection process

Statistical heterogeneity was assessed using Higgins *I*^2^ statistic for each pooled analysis. This was interpreted in accordance with Higgins and Green [22]. A random effects model was chosen owing to multiple analyses carrying a Higgins I^2^ > 75%, which represents considerable statistical heterogeneity. Further, we used a random-effects model to account for the potential unknown variability which may occur when pooling patient populations from various different continents. Variables not included in the meta-analysis were synthesized in a combination of descriptive and narrative analyses (Fig. 1).

## Results

A total of 4,972 records were screened, with 126 potentially eligible articles identified (Fig. 1). Sixteen were excluded on the basis of the pre-specified exclusion criteria. A total of 110 studies evaluating 115,496 patients were included. Twenty-one studies did not report on the number of knees included [23–43]. Ninety-four studies reported patient sex (38,134 males, 33.4%, and 76,073 females, 66.6%). Patient age ranged from six to 83 years. The United States (24 studies), China (23 studies), and the United Kingdom (10 studies) were the most represented countries (Table 1).

### Study quality assessment

The findings of the study quality assessment are presented in Appendix 2. Risk of bias could not be assessed in eight studies due to these being non-full text articles [27, 30, 44–49]. Risk of bias was deemed low in three studies only [40, 50, 51]. All case series included exhibited a high risk of bias. Overall, the majority of studies included in this review exhibited methodological limitations in terms of low level of evidence and risk of bias (Appendix 2). Visual assessment of funnel plots revealed asymmetries indicative of small study bias for all analyses conducted (Appendix 3).

### Meta-analysis

The results of the meta-analysis can be found in Table 2. Overlapping confidence intervals observed in analyses including all studies and those excluding outliers based on sample size suggest no statistically significant difference between both.

**Table 2:** A total of 76 studies reported that patellar fracture did not occur in the study population, with 34 studies reporting at least a single case of patellar fracture. The incidence of patellar fracture ranged from 0 to 9.50%, with a pooled incidence of 1.17% (95% CI 0.76–1.68%; *I*^2^ 94.28%). Meta-analysis results

Group	Range of fracture incidence (%)	Pooled incidence (%)	95% CI (%)	Higgins I^2^ (%)	Cochran’s Q	Number of patients	Number of studies
Meta-analyses							
Fracture incidence	0–9.50	1.17	0.76–1.68	94.28	1906	115,496	110
Fracture incidence in paediatric population	0–4.0	1.44	0.53–2.75	0	6.95	468	13
Fracture incidence in adult population	0–1.72	1.38	0.24–3.28	0	0.66	250	7
Allograft	0–5	1.78	0.75–3.22	0	10.08	458	13
Gracilis tendon autograft	0–3.90	1.06	0.48–1.85	0	15.97	898	17
Semitendinosus tendon autograft	0–7.14	1.00	0.55–1.58	0	17.00	1469	42
Synthetic graft	0–3.33	1.44	0–6.5	59.00	2.44	115	2
Tunnels drilled	0–7.14	0.94	0.69–1.24	0	62.88	4818	88
No tunnels drilled	0–2	1.00	0.15–2.44	0	1.66	323	7
Both femoral and patellar tunnels drilled	0–5.88	1.05	0.65–1.53	0	34.50	2121	37
Femoral tunnel drilled	0–7.14	1.05	0.50–1.78	0	9.79	1004	32
Patellar tunnel drilled	0–3.51	1.02	0.45–1.81	21.11	24.08	1539	20
No concomitant procedures	0–7.14	0.86	0.54–1.26	0	24.42	2546	47
Underwent concomitant procedures	0–9.50	1.26	0.51–2.32	87.43	493.24	9663	58
Concomitant tibial tubercle osteotomy	0–3.13	0.49	0.10–1.12	0	2.66	833	9
Sensitivity analyses							
Fracture incidence	0–7.14	0.85	0.63–1.11	0	63.55	5734	65
Fracture incidence in paediatric population	0–3.51	1.05	0.18–2.50	0	3.65	333	6
Fracture incidence in adult population	0–1.72	1.44	0.11–3.86	0	0.41	177	3
Allograft	0–4.35	1.40	0.35–3.04	12.78	6.89	368	7
Gracilis tendon autograft	0–3.90	0.98	0.31–1.99	19.56	13.67	798	12
Semitendinosus tendon autograft	0–7.14	0.77	0.31–1.42	0	10.43	999	16
Synthetic graft	NA	NA	NA	NA	NA	NA	NA
Tunnels drilled	0–7.14	0.91	0.63–1.25	7.38	57.22	4149	54
No tunnels drilled	0–2	0.93	0.04–2.61	0	1.50	247	3
Both femoral and patellar tunnels drilled	0–5.88	1.02	0.54–1.64	22.63	31.02	1850	25
Femoral tunnel drilled	0–7.14	0.96	0.31–1.90	0	9.05	615	12
Patellar tunnel drilled	0–3.51	0.88	0.33–1.69	28.61	16.81	1430	13
No concomitant procedures	0–7.14	0.75	0.42–1.16	0	18.29	2116	25
Underwent concomitant procedures	0–5.88	0.85	0.54–1.24	8.20	40.30	2992	33
Concomitant tibial tubercle osteotomy	0–3.13	0.53	0.13–1.16	0	3.63	802	6

#### Incidence

A total of 76 studies reported that patellar fracture did not occur in the study population, with 34 studies reporting at least a single case of patellar fracture (Appendix 4). The incidence of patellar fracture ranged from 0 to 9.50%, with a pooled incidence of 1.17% (95% CI 0.76–1.68%; *I*^2^ 94.28%). A sensitivity analysis performed by removing outliers in sample size revealed an incidence range of 0 to 7.14%, and a pooled incidence of 0.85% (95% CI 0.63–1.11%; *I*^2^ 0%).

#### Patient age

Thirteen studies reported on exclusively paediatric populations for meta-analysis (*n* = 468).

 [34, 37, 48, 52–61]. Incidence of patellar fractures ranged between 0 and 4.0%. Seven studies reported on MPFLR in adult populations (*n* = 250) [24, 47, 62–66], with reported incidence of patellar fractures between 0 and 1.72%. There was no difference in the incidence of patellar fractures between both age groups, with an incidence of 1.44% among children (95% CI 0.53–2.75%, *I*^2^ 0%) and of 1.38% among adults (95% CI 0.24–3.28%, *I*^2^ 0%).

#### Use of bone tunnels

A total of 88 studies reported utilising tunnels for meta-analysis (*n* = 4,818). Incidence of patellar fractures among these ranged from 0 to 7.14%, with a pooled incidence of 0.94% (95% CI 0.69–1.24%, *I*^2^ 0%). There was no statistically significant difference in incidence of fractures compared to studies which did not drill bone tunnels (1.00%, 95% CI 0.15–2.44%, *I*^2^: 0%), with seven studies pooled in this analysis (*n* = 323) [36, 44, 59, 60, 67–69]. A similar incidence of patellar fractures was observed in studies which drilled both femoral and patellar tunnels (1.05%, 95% CI 0.65–1.53%, *I*^2^: 0%), femoral tunnels only (1.05%, 95% CI 0.50–1.78%, *I*^2^: 0%), or patellar tunnels only (1.02%, 95% CI 0.45–1.81%, *I*^2^: 21.11%).

#### Graft type used

No fractures were reported in studies utilising peroneus longus tendon autograft (*n* = 33) [61, 70, 71] or quadriceps autograft (*n* = 190) [38, 39, 60, 71–76]. Other types of grafts had higher incidences of fracture, though these did not statistically significantly differ between each other. These were allograft (1.78%, 95% CI 0.75–3.22%, *I*^2^: 0%), gracilis tendon autograft (1.06%, 95% CI 0.48–1.85%, *I*^2^: 0%), semitendinosus tendon autograft (1.00%, 95% CI 0.55–1.58%, *I*^2^: 0%), and synthetic grafts (1.44%, 95% CI 0.0–6.50%, *I*^2^: 59%). The anchoring methods varied between studies, and are described in Appendix 4.

#### Performance of concomitant procedures

A total of 58 studies performed concomitant procedures (*n* = 9,663), whereas 47 did not (*n* = 2,546). There was no statistically significant difference in the incidence of fractures between these groups. Studies performing concomitant procedures had a pooled fracture incidence of 1.26% (95% CI 0.51–2.32%, *I*^2^: 87.43%), compared to an incidence of 0.86% (95% CI 0.54–1.26%, *I*^2^: 0%) in studies performing MPFLR alone. No fractures were reported in studies performing concomitant chondroplasty (*n* = 31) [59, 60, 71, 73, 77, 78], lateral retinacular release (*n* = 157) [55, 59, 65, 70, 77–81], loose body removal (*n* = 8) [59, 60, 82], and meniscal repair (*n* = 10) [59, 60, 79, 82]. Nine studies reported on concomitant tibial tubercle osteotomy (TTO) for meta-analysis (*n* = 833) [36, 47, 50, 65, 83–87]. Patients undergoing TTO had a pooled incidence of post-operative patellar fracture of 0.49% (95% 0.10–1.12%, *I*^2^: 0%).

### Narrative synthesis

#### Time of patellar fracture

Time elapsed between operation and fracture occurrence varied among the 13 studies reporting on this (Table 3), with fractures occurring as early as four weeks [44]. Notably, an analysis of 29,539 patients carried out by Fitzgerald et al. found that 31.9% of fractures occurred in the first 90 days, 36.9% occurred between 90 days and one year, and 38.3% occurred 1 year after surgery [27].

**Table 3:** Time elapsed between operation and fracture occurrence varied among the 13 studies reporting on this, with fractures occurring as early as four weeks. Time of fracture occurrence

Study	Number of patients	Number of fractures	Time of fracture
Allahabadi and Pandya [101]	20	1	6 weeks
Astur et al. [24]	58	1	Before 2 years
Christiansen et al. [93]	44	1	6 weeks
Fitzgerald et al. [27]	29,539	141	31.9% of fractures occurred in the first 90 days, 36.9% occurring between 90 days and 1 year, 38.3% occurring after 1 year
Hohn and Pandya [53]	25	1	6 months
Howells and Eldridge [51]	75	1	6 months
Kita et al. [90]	42	3	2, 4, and 9 months
Kodkani et al. [67]	50	1	8 months
Mikashima et al. [44]	24	2	4 and 5 weeks
Moran et al. [85]	560	1	4 months
Panni et al. [92]	48	**1**	4 months
Schiphouwer et al. [124]	179	6	Mean: 5.8 months (±4.3)
Deasey et al. [84]	352	1	16 weeks

#### Mechanism of patellar fracture

Eleven studies reported on fracture mechanism. Falls were the most common cause of fractures, reported in seven studies [44, 51, 53, 67, 88–90]. One study attributed a case of fracture to early return to sports [91], whereas Panni et al. reported the case of fracture was due to direct trauma (mechanism unspecified) [92]. Two studies attributed fractures to surgical error. Christiansen et al. described a case in which the patellar tunnel breached the anterior cortex, with the fracture occurring when the patient rose from a chair [93]. Similarly, Hopper et al. stated that four fractures occurred due to the patellar tunnel being placed too anteriorly [94].

#### Treatment and outcomes of patellar fractures

Fifteen studies reported on the treatment of patellar fractures (Table 4). Open reduction and internal fixation (ORIF) was the most common treatment, followed by non-operative management. Of nine studies reporting on outcomes, six reported achieving union, with the remaining three not reporting on this specifically. Three studies reported that transverse fractures occurred [48, 67, 92], whereas Parikh et al. reported that fractures occurred through the tunnel [95]. A single study provided details of the ORIF performed, reporting the use of cannulated, cancellous screws and cerclage wires [90]. Only one patient experienced complications associated with patellar fracture repair, with Kodkani et al. reporting a case of terminal flexion limited to 110° and mild thigh muscle atrophy, with otherwise near normal knee function [67].

**Table 4:** Fifteen studies reported on the treatment of patellar fractures. Open reduction and internal fixation was the most common treatment, followed by non-operative management. Of nine studies reporting on outcomes, six reported achieving union, with the remaining three not reporting on this specifically. Mechanism and treatment of patellar fractures

Study	Number of patients	Number of fractures	Treatment	Type fracture and treatment details	Outcomes
Allahabadi and Pandya [101]	20	1	ORIF	NR	NR
Astur et al. [24]	58	1	Screw fixation	NR	“Good results” reported
Hopper et al. [94]	68	4	ORIF or screw fixation	NR	Union
Howells et al. [89]	193	1	Non-operatively	NR	Union
Howells and Eldridge [51]	75	1	Non-operatively	NR	Union
Kim et al. [32]	81	1	Cast application for 4 weeks (nondisplaced fracture)	NR	NR
Kita et al. [91]	24	1	ORIF	NR	NR
Kita et al. [90]	42	3	ORIF	Performed with the use of cannulated, cancellous screws and cerclage wires	Union
Kodkani et al. [67]	50	1	ORIF + K wires and tension band wiring	Transverse fracture of the patella	Terminal flexion limited to ~110° and mild thigh muscle atrophy. Her knee function was near normal (Kujala score 91 at 1-year post-fracture).
Mikashima et al. [44]	24	2	Case 1: patellar fragment removal. Case 2: rehabilitation	NR	Both recovered successfully
Mulliez et al. [68]	124	1	Revision MPFL	NR	NR
Panni et al. [92]	48	1	Two partially threaded cannulated screws and immobilization	Transverse fracture of the patella	Union
Parikh et al. [95]	154	6	4 tension-band wiring, 1 cast immobilisation, 1 quadriceps-tendon repair	5 transverse fracture through the tunnel, one non-displaced transverse fracture	Union
Reddy et al. [57]	57	2	Tension band wiring	NR	NR
Yoon et al. [48]	46	2	ORIF	Transverse fracture	NR
*ORIF* open reduction internal fixation, *NR* not reported					

## Discussion

We identified an incidence of patellar fracture following MPFLR of 1.17% (95% CI 0.76–1.68%), ranging from 0 to 9.50% among included studies. Knowledge of this may allow health professionals to hold informed discussions with patients and relatives regarding prognosis following MPFLR, and emphasises the importance of post-operative follow-up. However, the majority of studies included in this review were case series which exhibited methodological limitations pertaining to low level of evidence and concerns regarding risk of bias. Caution should therefore be placed when interpreting these findings.

The incidence of patellar fractures in the general population has been reported as 0.01% per year [96]. Our calculated fracture incidence following MPFLR of 1.17% is much higher than the baseline risk. This discrepancy may reflect the iatrogenic risk following surgery. The creation of transosseous tunnels or the placement of large suture anchors may create stress within the bone, weakening the patella’s structural integrity [84]. However, it is possible that patients with patellar instability are at a higher risk of fractures due to altered patellofemoral kinematics or reduced bone density due to less activity [97]. Therefore, the isolated effect of MPFLR on fracture risk is not clear. Further cohort or case-control studies are required to compare fracture risk in patients with patellofemoral instability who did or did not undergo MPFLR.

We did not identify an increased incidence of post-operative fractures among paediatric or adult populations. This suggests that, despite the higher prevalence of patellar instability among children and adolescents [98], this age group does not have a worse prognosis than adults when it comes to the risk of suffering from a post-operative patellar fracture. In addition, drilling of bone tunnels or performing concomitant procedures were not associated with a higher incidence of patellar fractures. However, the lack of statistically significant differences observed between groups may be attributed to the low incidence of patellar fractures, which may have yielded a statistically under-powered analysis of these parameters. Meta-analyses of odds or risk ratios may be better suited to establish the association between risk of patellar fractures and patient demographics and surgical technique.

No differences in the incidence of patellar fractures were observed between studies which utilised allograft, synthetic grafts, or gracilis or semitendinosus tendon autograft. No fractures were reported in studies utilising peroneus longus tendon autograft or quadriceps autograft. However, there is no sufficient evidence to suggest that these grafts are associated with a low incidence of patellar fractures, as studies reporting on these amounted to a combined 33 and 190 patients, respectively. Similarly, though no studies reported the occurrence of patellar fracture following chondroplasty (*n* = 31), lateral retinacular release (*n* = 157), loose body removal (*n* = 8), and meniscal repair (*n* = 10), the low sample sizes for each procedure hinders the claim that these studies are not associated with post-operative patellar fractures.

Time elapsed between operation and fracture occurrence varied among the 13 studies reporting on this, with fractures occurring as early as four weeks [44]. No studies reported fractures occurring more than two years after MPFLR. An analysis of 29,539 patients carried out by Fitzgerald et al. found that 31.9% of fractures occurred in the first 90 days, 36.9% occurred between 90 days and one year, and 38.3% occurred 1 year after surgery, but did not report when the latest fracture occurred [27]. Surgeons may expect post-operative fractures to occur within two years of surgery, though further studies reporting on time of fracture are required to ascertain this.

Eleven studies reported on fracture mechanism. Falls were the most common cause of fractures, reported in seven studies. Two studies attributed fractures to surgical error, with both describing the patellar tunnel being placed too anteriorly as the cause of fracture [93, 94]. This suggests that correct anatomical positioning is required to mitigate the risk of fractures. Fifteen studies reported on the treatment of patellar fractures, with open reduction and internal fixation being the most common corrective procedure. However, only nine studies reported on outcomes, with one study reporting a case of limited terminal flexion and thigh muscle atrophy following surgical repair [67]. There is therefore limited evidence to ascertain the mechanism and outcomes of patellar fracture following MPFLR.

Current evidence has limitations which must be considered when interpreting a study’s findings. First, we included studies which explicitly stated whether patellar fractures occurred. It is possible that we have overestimated the incidence of patellar fractures by virtue of multiple studies in the literature not reporting on the occurrence of this complication. Though analysis of funnel plots revealed publication bias for all parameters evaluated, this may be attributed to patellar fractures being a rare complication and therefore unlikely to be reported in small case series, rather than to publication bias. Second, the majority of studies included in this review were case series which exhibited methodological limitations pertaining to low level of evidence and concerns regarding risk of bias. Third, a limited number of studies were prospective in design, which hinders the establishment of a cause-and-effect relationship between MPFLR and patellar fractures. Fourth, no studies reported on the ethnicity of included patients, which limits the ability to evaluate inequalities in the outcomes of MPFLR. Finally, there was a low number of studies reporting on the mechanism (11, 10%), treatment and outcomes (15, 13.6%) of patellar fractures. Though previous meta-analyses comparing surgical interventions for patellar fractures have been performed [99, 100], fractures of the patella following MPFLR may present with different kinematics. Further work on these is required to provide a robust evidence base on the management of patellar fractures occurring after MPFLR.

## Conclusion

Patellar fractures are a common complication of MPFLR, with a higher incidence observed in patients undergoing this procedure compared to the baseline population risk. Current evidence suggests its incidence is not affected by tunnel location. Appropriate post-operative follow-up is required to monitor the occurrence of patellar fractures during the post-operative period.

## Data Availability

No datasets were generated or analysed during the current study.

## Appendix 1: search strategy

1. MPFL OR medial patellofemoral ligament
2. Repair OR surgery OR reconstruction OR operation OR intervention OR procedure
3. 1 AND 2
4. MPFLR
5. 3 OR 4

Deduplicate.

## Appendix 2

See Tables 5

**Table 5:** Risk of bias assessment

Institute of health economics case series quality appraisal checklist [16]	Allahabadi et al. 2022 [101]	Ambrožič and Novak, 2016 [72]	Arshi et al. 2016 [23]	Bachman et al. 2024 [52]	Chatterton et al. 2018 [83]	Christiansen et al. 2008 [93]	Deie et al. 2011 [102]	Deo et al. 2023 [103]	Devgan et al. 2019 [104]
Was the hypothesis/aim/objective of the study clearly stated?	Yes	Yes	Yes	Yes	Yes	Yes	Yes	Yes	Yes
Was the study conducted prospectively?	No	No	No	No	No	Yes	Unclear	No	Yes
Were the cases collected in more than one centre?	No	No	Yes	Yes	No	No	No	No	No
Were patients recruited consecutively?	Unclear	Unclear	Unclear	Unclear	Unclear	Unclear	Unclear	Unclear	Unclear
Were the characteristics of the patients included in the study described?	Yes	Yes	Yes	Yes	Yes	Yes	Yes	Yes	Yes
Were the eligibility criteria (i.e., inclusion and exclusion criteria) for entry into the study clearly stated?	Yes	Yes	Yes	Yes	Yes	Yes	Yes	Yes	Yes
Did patients enter the study at a similar point in the disease?	Yes	Yes	Yes	Yes	Yes	Yes	Yes	Yes	Yes
Was the intervention of interest clearly described?	Yes	Yes	Yes	Yes	Yes	Yes	Yes	Yes	Yes
Were additional interventions (co-interventions) clearly described?	Yes	Yes	Yes	Yes	Yes	Yes	Yes	Yes	Unclear
Were relevant outcome measures established a priori?	Unclear	Unclear	Unclear	Unclear	Unclear	Unclear	Unclear	Unclear	Unclear
Were outcome assessors blinded to the intervention that patients received?	Unclear	Unclear	Unclear	Unclear	Unclear	Unclear	Unclear	Unclear	Unclear
Were the relevant outcomes measured using appropriate objective/subjective methods?	Yes	Yes	Yes	Yes	Yes	Yes	Yes	Yes	Yes
Were the statistical tests used to assess the relevant outcomes appropriate?	Yes	Yes	Yes	Yes	Yes	Yes	Yes	Yes	No
Was follow-up long enough for important events and outcomes to occur?	Yes	Yes	NA	Yes	No	Yes	Yes	Yes	Yes
Were losses to follow-up reported?	Yes	Yes	NA	Yes	Yes	Yes	Yes	Yes	Yes
Did the study provide estimates of random variability in the data analysis of relevant outcomes?	Yes	Yes	Yes	Yes	Yes	Yes	Yes	Yes	Yes
Were the adverse events reported?	Yes	Yes	Yes	Yes	Yes	Yes	Yes	Yes	Yes
Were the conclusions of the study supported by results?	Yes	Yes	Yes	Yes	Yes	Yes	Yes	Yes	Yes
Were both competing interests and sources of support for the study reported?	Yes	Yes	Yes	Yes	No	Yes	Yes	Yes	Yes
Global Judgement	High	High	High	High	High	High	High	High	High

Institute of health economics case series quality appraisal checklist [16]	Dong et al. 2023 [105]	Enderlein et al. 2014 [106]	Fadel and Hosni, 2020 [71]	Fisher et al. 2021 [73]	Fuji et al. 2021 [107]	Gomes, 1992 [88]	Gorbaty et al. 2022 [29]	Goyal et al. 2018 [62]	Han et al. 2011 [109]	Hohn et al. 2017 [53]
Was the hypothesis/aim/objective of the study clearly stated?	Yes	Yes	Yes	Yes	Yes	Yes	Yes	Yes	Yes	Yes
Was the study conducted prospectively?	Yes	Yes	Yes	No	No	Unclear	No	No	No	No
Were the cases collected in more than one centre?	No	No	No	No	No	Unclear	Yes	No	No	No
Were patients recruited consecutively?	Unclear	Unclear	Unclear	Unclear	Unclear	Unclear	Unclear	Unclear	Unclear	Unclear
Were the characteristics of the patients included in the study described?	Yes	Yes	Yes	Yes	Yes	Yes	Yes	Yes	Yes	Yes
Were the eligibility criteria (i.e., inclusion and exclusion criteria) for entry into the study clearly stated?	Yes	Yes	Yes	Yes	Yes	No	Yes	Yes	Yes	Yes
Did patients enter the study at a similar point in the disease?	Yes	Yes	Yes	Yes	Yes	Yes	Yes	Yes	Yes	Yes
Was the intervention of interest clearly described?	Yes	Yes	Yes	Yes	Yes	Yes	Yes	Yes	Yes	Yes
Were additional interventions (co-interventions) clearly described?	Yes	Yes	Yes	Yes	Yes	No	Yes	Yes	Yes	Yes
Were relevant outcome measures established a priori?	Unclear	Unclear	Unclear	Unclear	Unclear	Unclear	Unclear	Unclear	Unclear	Unclear
Were outcome assessors blinded to the intervention that patients received?	Unclear	Unclear	Unclear	Unclear	Unclear	Unclear	Unclear	Unclear	Unclear	Unclear
Were the relevant outcomes measured using appropriate objective/subjective methods?	Yes	Yes	Yes	Yes	Yes	No	Yes	No	Yes	Yes
Were the statistical tests used to assess the relevant outcomes appropriate?	Yes	Yes	Yes	Yes	Yes	No	Yes	No	Yes	No
Was follow-up long enough for important events and outcomes to occur?	Yes	Yes	Yes	Yes	Yes	No	NA	Yes	Yes	Yes
Were losses to follow-up reported?	Yes	Yes	Yes	Yes	Yes	No	No	No	Yes	Yes
Did the study provide estimates of random variability in the data analysis of relevant outcomes?	Yes	Yes	Yes	Yes	Yes	No	No	No	Yes	No
Were the adverse events reported?	Yes	Yes	Yes	Yes	Yes	No	Yes	Yes	Yes	Yes
Were the conclusions of the study supported by results?	Yes	Yes	Yes	Yes	Yes	Yes	Yes	Yes	Yes	Yes
Were both competing interests and sources of support for the study reported?	Yes	No	No	Yes	No	Yes	Yes	Yes	No	Yes
Global Judgement	High	High	High	High	High	High	High	High	High	High

Institute of health economics case series quality appraisal checklist [16]	Hong et al. 2024 [77]	Hopper et al. 2014 [94]	Howells et al. 2012 [89]	Kang et al. 2014 [31]	Kita et al. 2012 [91]	Kita et al. 2017 [113]	Kodkani et al. 2016 [67]	Kumahashi et al. 2014 [114]	Kumahashi et al. 2012 [55]	Kumar et al. 2014 [9]
Was the hypothesis/aim/objective of the study clearly stated?	Yes	Yes	Yes	Yes	Yes	Yes	Yes	Yes	Yes	Yes
Was the study conducted prospectively?	Yes	No	Yes	No	Unclear	Unclear	Yes	No	No	No
Were the cases collected in more than one centre?	No	Yes	No	No	No	No	No	No	No	No
Were patients recruited consecutively?	Unclear	Unclear	Unclear	Unclear	Unclear	Unclear	Unclear	Unclear	Unclear	Unclear
Were the characteristics of the patients included in the study described?	Yes	Yes	Yes	Yes	Yes	Yes	Yes	Yes	Yes	Yes
Were the eligibility criteria (i.e., inclusion and exclusion criteria) for entry into the study clearly stated?	Yes	No	No	Yes	Yes	Yes	Yes	Yes	Yes	Yes
Did patients enter the study at a similar point in the disease?	Yes	Yes	Yes	Yes	Yes	Yes	Yes	Yes	Yes	Yes
Was the intervention of interest clearly described?	Yes	Yes	Yes	Yes	Yes	Yes	Yes	Yes	Yes	Yes
Were additional interventions (co-interventions) clearly described?	Yes	Yes	No	Yes	No	Yes	Yes	Yes	Yes	Yes
Were relevant outcome measures established a priori?	Unclear	Unclear	Unclear	Unclear	Unclear	Unclear	Unclear	Unclear	Unclear	Unclear
Were outcome assessors blinded to the intervention that patients received?	Unclear	Unclear	Unclear	Unclear	Unclear	Unclear	Unclear	Unclear	Unclear	Unclear
Were the relevant outcomes measured using appropriate objective/subjective methods?	Yes	Yes	Yes	Yes	Yes	Yes	Yes	Yes	Yes	Yes
Were the statistical tests used to assess the relevant outcomes appropriate?	Yes	Yes	Yes	Yes	Yes	Yes	No	Yes	Yes	Yes
Was follow-up long enough for important events and outcomes to occur?	Yes	Yes	Yes	Yes	Yes	Yes	No	Yes	Yes	Yes
Were losses to follow-up reported?	Yes	Yes	No	Yes	No	No	No	No	No	No
Did the study provide estimates of random variability in the data analysis of relevant outcomes?	Yes	Yes	Yes	Yes	Yes	Yes	No	Yes	Yes	No
Were the adverse events reported?	Yes	Yes	Yes	Yes	Yes	Yes	Yes	Yes	Yes	Yes
Were the conclusions of the study supported by results?	Yes	Yes	Yes	Yes	Yes	Yes	Yes	Yes	Yes	Yes
Were both competing interests and sources of support for the study reported?	Yes	Yes	No	Yes	Yes	Yes	Yes	Yes	No	Yes
Global Judgement	High	High	High	High	High	High	High	High	High	High

Institute of health economics case series quality appraisal checklist [16]	Kumar et al. 2018 [56]	Lin et al. 2015 [115]	Liu et al. 2018 [79]	Maione et al. 2023 [116]	Marigi et al. 2023 [117]	Matthews et al. 2010 [118]	Mochizuki et al. 2019 [82]	Mohammed et al. 2017 [119]	Monllau et al. 2016 [120]	Mulliez et al. 2017 [68]
Was the hypothesis/aim/objective of the study clearly stated?	Yes	Yes	Yes	Yes	Yes	Yes	Yes	Yes	Yes	Yes
Was the study conducted prospectively?	No	Yes	No	No	No	Unclear	Unclear	No	Yes	Yes
Were the cases collected in more than one centre?	No	No	No	No	Yes	No	No	Yes	No	No
Were patients recruited consecutively?	Unclear	Unclear	Unclear	Unclear	Unclear	Unclear	Unclear	Unclear	Unclear	Unclear
Were the characteristics of the patients included in the study described?	Yes	Yes	Yes	Yes	Yes	Yes	Yes	Yes	Yes	Yes
Were the eligibility criteria (i.e., inclusion and exclusion criteria) for entry into the study clearly stated?	Yes	Yes	Yes	Yes	Yes	Yes	Yes	Yes	Yes	Yes
Did patients enter the study at a similar point in the disease?	Yes	Yes	Yes	Yes	Yes	Yes	Yes	Yes	Yes	Yes
Was the intervention of interest clearly described?	Yes	Yes	Yes	Yes	Yes	Yes	Yes	Yes	Yes	Yes
Were additional interventions (co-interventions) clearly described?	Yes	Yes	Yes	Yes	Yes	Yes	Yes	Yes	Yes	Yes
Were relevant outcome measures established a priori?	Unclear	Unclear	Unclear	Unclear	Unclear	Unclear	Unclear	Unclear	Unclear	Unclear
Were outcome assessors blinded to the intervention that patients received?	Unclear	Unclear	Unclear	Unclear	Unclear	Unclear	Unclear	Unclear	Unclear	Unclear
Were the relevant outcomes measured using appropriate objective/subjective methods?	Yes	Yes	Yes	Yes	Yes	Yes	Yes	No	Yes	Yes
Were the statistical tests used to assess the relevant outcomes appropriate?	Yes	Yes	Yes	Yes	Yes	No	Yes	Yes	Yes	Yes
Was follow-up long enough for important events and outcomes to occur?	Yes	Yes	Yes	Yes	Yes	Yes	Yes	Yes	No	Yes
Were losses to follow-up reported?	No	Yes	Yes	Yes	Yes	No	No	No	Yes	Yes
Did the study provide estimates of random variability in the data analysis of relevant outcomes?	No	Yes	No	Yes	Yes	No	Yes	No	Yes	No
Were the adverse events reported?	Yes	Yes	Yes	Yes	Yes	Yes	Yes	Yes	Yes	Yes
Were the conclusions of the study supported by results?	Yes	Yes	Yes	Yes	Yes	Yes	Yes	Yes	Yes	Yes
Were both competing interests and sources of support for the study reported?	Yes	Yes	Yes	Yes	Yes	No	Yes	Yes	No	No
Global Judgement	High	High	High	High	High	High	High	High	High	High

Institute of health economics case series quality appraisal checklist [16]	Nazeer et al. 2023 [69]	Niu et al. 2017 [121]	Ntagiopoulos et al. 2024 [34]	Özel et al. 2025 [123]	Palacios et al. 2017 [35]	Panni et al. 2011 [92]	Parikh et al. 2013 [95]	Park et al. 2025 [36]	Perkins et al. 2021 [37]	Reddy et al. 2022 [55]
Was the hypothesis/aim/objective of the study clearly stated?	Yes	Yes	Yes	Yes	Yes	Yes	Yes	Yes	Yes	Yes
Was the study conducted prospectively?	No	Yes	No	No	No	Yes	No	No	No	No
Were the cases collected in more than one centre?	No	No	No	No	No	No	No	Yes	No	No
Were patients recruited consecutively?	Unclear	Unclear	Unclear	Unclear	Unclear	Unclear	Unclear	Unclear	Unclear	Unclear
Were the characteristics of the patients included in the study described?	Yes	Yes	Yes	Yes	Yes	Yes	Yes	Yes	Yes	Yes
Were the eligibility criteria (i.e., inclusion and exclusion criteria) for entry into the study clearly stated?	Yes	Yes	Yes	Yes	Yes	Yes	Yes	Yes	Yes	Yes
Did patients enter the study at a similar point in the disease?	Yes	Yes	Yes	Yes	Yes	Yes	Yes	Yes	Yes	Yes
Was the intervention of interest clearly described?	Yes	Yes	Yes	Yes	Yes	Yes	Yes	Yes	Yes	Yes
Were additional interventions (co-interventions) clearly described?	Yes	Yes	Yes	No	No	No	No	No	No	No
Were relevant outcome measures established a priori?	Unclear	Unclear	Unclear	Unclear	Unclear	Unclear	Unclear	Unclear	Unclear	Unclear
Were outcome assessors blinded to the intervention that patients received?	Unclear	Unclear	Unclear	Unclear	Unclear	Unclear	Unclear	Unclear	Unclear	Unclear
Were the relevant outcomes measured using appropriate objective/subjective methods?	Yes	Yes	Yes	Yes	Yes	Yes	Yes	Yes	Yes	Yes
Were the statistical tests used to assess the relevant outcomes appropriate?	No	Yes	Yes	Yes	No	Yes	Yes	Yes	Yes	Yes
Was follow-up long enough for important events and outcomes to occur?	Yes	Yes	Yes	Yes	Yes	Yes	Yes	No	Yes	Yes
Were losses to follow-up reported?	Yes	Yes	Yes	Unclear	Unclear	Unclear	Unclear	Unclear	Unclear	Unclear
Did the study provide estimates of random variability in the data analysis of relevant outcomes?	No	Yes	Yes	Yes	Yes	No	No	No	No	No
Were the adverse events reported?	Yes	Yes	Yes	No	Yes	Yes	Yes	Yes	Yes	Yes
Were the conclusions of the study supported by results?	Yes	Yes	Yes	Yes	Yes	Yes	Yes	Yes	Yes	Yes
Were both competing interests and sources of support for the study reported?	Yes	Yes	Yes	Yes	Yes	Yes	Yes	Yes	Yes	Yes
Global Judgement	High	High	High	High	High	High	High	High	High	High

Institute of health economics case series quality appraisal checklist [16]	Rhatomy et al. 2019 [38]	Ronga et al. 2009 [63]	Sanguanjit et al. 2023 [39]	Sim et al. 2018 [125]	Song et al. 2019 [64]	Song et al. 2014 [78]	Steiner et al. 2006 [75]	Sundararajan et al. 2015 [41]	Tang et al. 2015 [126]	Teküstün et al. 2025 [76]
Was the hypothesis/aim/objective of the study clearly stated?	Yes	Yes	Yes	Yes	Yes	Yes	Yes	Yes	Yes	Yes
Was the study conducted prospectively?	No	No	Yes	No	No	Unclear	No	Yes	Unclear	No
Were the cases collected in more than one centre?	No	No	No	No	No	No	No	No	No	No
Were patients recruited consecutively?	Unclear	Unclear	Unclear	Unclear	Unclear	Unclear	Unclear	Unclear	Unclear	Unclear
Were the characteristics of the patients included in the study described?	Yes	Yes	Yes	Yes	Yes	Yes	Yes	Yes	Yes	Yes
Were the eligibility criteria (i.e., inclusion and exclusion criteria) for entry into the study clearly stated?	Yes	Yes	Yes	Yes	Yes	Yes	Yes	Yes	Yes	Yes
Did patients enter the study at a similar point in the disease?	Yes	Yes	Yes	Yes	Yes	Yes	Yes	Unclear	No	Yes
Was the intervention of interest clearly described?	Yes	Yes	Yes	Yes	Yes	Yes	Yes	Yes	Yes	Yes
Were additional interventions (co-interventions) clearly described?	No	No	No	No	No	No	No	No	No	No
Were relevant outcome measures established a priori?	Unclear	Unclear	Unclear	Unclear	Unclear	Unclear	Unclear	Unclear	Unclear	Unclear
Were outcome assessors blinded to the intervention that patients received?	Unclear	Unclear	Unclear	Unclear	Unclear	Unclear	Unclear	Unclear	Unclear	Unclear
Were the relevant outcomes measured using appropriate objective/subjective methods?	Yes	Yes	Yes	Yes	Yes	Yes	Yes	Yes	Yes	Yes
Were the statistical tests used to assess the relevant outcomes appropriate?	Yes	Yes	Yes	Yes	Yes	Yes	Yes	Yes	Yes	Yes
Was follow-up long enough for important events and outcomes to occur?	Yes	Yes	Yes	Yes	Yes	Yes	Yes	Partial	Yes	Yes
Were losses to follow-up reported?	Unclear	Yes	Yes	Yes	No	Yes	Yes	Yes	Yes	NA
Did the study provide estimates of random variability in the data analysis of relevant outcomes?	No	Yes	Yes	Yes	No	No	Yes	No	Partial	No
Were the adverse events reported?	Yes	Yes	Yes	Yes	Yes	Yes	Yes	Yes	Yes	Yes
Were the conclusions of the study supported by results?	Yes	Yes	Yes	Yes	Yes	Yes	Yes	Yes	Yes	Yes
Were both competing interests and sources of support for the study reported?	Yes	Yes	Yes	Yes	Yes	Yes	No	Yes	No	Yes
Global Judgement	High	High	High	High	High	High	High	High	High	High

Institute of health economics case series quality appraisal checklist [16]	Toritsuka et al. 2011 [127]	Torkaman et al. 2015 [42]	Valkering et al. 2017 [43]	van Gennip et al. 2014 [65]	von Engelhardt et al. 2017 [87]	Wang et al. 2016 [128]	Wu et al. 2025 [129]	Xing et al. 2022 [130]	Yang et al. 2017 [70]	Yoon et al. 2024 [131]
Was the hypothesis/aim/objective of the study clearly stated?	Yes	Yes	Yes	Yes	Yes	Yes	Yes	Yes	Yes	Yes
Was the study conducted prospectively?	No	No	No	No	No	No	No	No	No	No
Were the cases collected in more than one centre?	No	No	No	No	No	No	No	No	No	No
Were patients recruited consecutively?	Unclear	Unclear	Unclear	Unclear	Unclear	Unclear	Unclear	Unclear	Unclear	Unclear
Were the characteristics of the patients included in the study described?	Yes	Yes	Yes	Yes	Yes	Yes	No	Yes	Yes	Yes
Were the eligibility criteria (i.e., inclusion and exclusion criteria) for entry into the study clearly stated?	Yes	Yes	Yes	Yes	Yes	Yes	Yes	Yes	Yes	Yes
Did patients enter the study at a similar point in the disease?	Unclear	Yes	Unclear	Unclear	Yes	Yes	Yes	Yes	Yes	Yes
Was the intervention of interest clearly described?	Yes	Yes	Yes	Yes	Yes	Yes	Yes	Yes	Yes	Yes
Were additional interventions (co-interventions) clearly described?	No	No	No	Yes	Yes	No	Yes	No	No	Yes
Were relevant outcome measures established a priori?	Unclear	Unclear	Unclear	Unclear	Unclear	Unclear	Unclear	Unclear	Unclear	Unclear
Were outcome assessors blinded to the intervention that patients received?	Unclear	Unclear	Unclear	Unclear	Unclear	Unclear	Unclear	Unclear	Unclear	Unclear
Were the relevant outcomes measured using appropriate objective/subjective methods?	Yes	Yes	Yes	Yes	Yes	Yes	Yes	Yes	Yes	Yes
Were the statistical tests used to assess the relevant outcomes appropriate?	Yes	Yes	Yes	Yes	Yes	Yes	Yes	Yes	Yes	Yes
Was follow-up long enough for important events and outcomes to occur?	Yes	Yes	Partial	Yes	Yes	Yes	Yes	Partial	Partial	Yes
Were losses to follow-up reported?	No	Yes	No	No	Unclear	Unclear	Yes	Yes	No	Yes
Did the study provide estimates of random variability in the data analysis of relevant outcomes?	Partial	Partial	Yes	Yes	Partial	Partial	No	Partial	No	Partial
Were the adverse events reported?	Partial	Yes	Partial	Partial	Yes	Yes	Yes	Yes	Partial	Yes
Were the conclusions of the study supported by results?	Yes	Yes	Yes	Yes	Yes	Yes	Yes	Yes	Yes	Yes
Were both competing interests and sources of support for the study reported?	Yes	Yes	Yes	Yes	Yes	Yes	Yes	Yes	Yes	Yes
Global Judgement	High	High	High	High	High	High	High	High	High	High

Institute of health economics case series quality appraisal checklist [16]	Yoon et al. 2020 [132]	Zein et al. 2024 [59]	Zein et al. 2024 [60]	Zhang et al. 2010 [133]	Zhang et al. 2023 [61]	Zhang et al. 2023 [134]	Zhou et al. 2014 [66]	Zhou et al. 2023 [135]	Zhu et al. 2017 [136]	Runer et al. 2023 [74]
Was the hypothesis/aim/objective of the study clearly stated?	Yes	Yes	Yes	Yes	Yes	Yes	Yes	Yes	Yes	Yes
Was the study conducted prospectively?	No	No	No	No	No	No	Unclear	No	Yes	No
Were the cases collected in more than one centre?	No	No	No	No	No	No	No	No	No	No
Were patients recruited consecutively?	Unclear	Unclear	Unclear	Unclear	Unclear	Unclear	Unclear	Unclear	Unclear	Unclear
Were the characteristics of the patients included in the study described?	Yes	Yes	Yes	Yes	Yes	Yes	Yes	Yes	Yes	Yes
Were the eligibility criteria (i.e., inclusion and exclusion criteria) for entry into the study clearly stated?	Yes	Yes	Yes	Yes	Yes	Yes	Yes	Yes	Yes	Yes
Did patients enter the study at a similar point in the disease?	Yes	Yes	Yes	No	Yes	Yes	Yes	Yes	Unclear	Yes
Was the intervention of interest clearly described?	Yes	Yes	Yes	Yes	Yes	Yes	Yes	Yes	Yes	Yes
Were additional interventions (co-interventions) clearly described?	Yes	No	No	No	Yes	No	No	No	No	Yes
Were relevant outcome measures established a priori?	Unclear	Unclear	Unclear	Unclear	Unclear	Unclear	Unclear	Unclear	Unclear	Unclear
Were outcome assessors blinded to the intervention that patients received?	Unclear	Unclear	Unclear	Unclear	Unclear	Unclear	Unclear	Unclear	Unclear	Unclear
Were the relevant outcomes measured using appropriate objective/subjective methods?	Yes	Yes	Yes	Yes	Yes	Yes	Yes	Yes	Yes	Yes
Were the statistical tests used to assess the relevant outcomes appropriate?	Yes	Yes	Yes	Yes	Yes	Yes	Yes	Yes	Yes	Yes
Was follow-up long enough for important events and outcomes to occur?	Yes	Yes	Yes	Yes	Yees	Yes	Yes	Yes	Yes	Yes
Were losses to follow-up reported?	Yes	Yes	NA	NA	No	No	Yes	No	No	NA
Did the study provide estimates of random variability in the data analysis of relevant outcomes?	Yes	Partial	Partial	Partial	No	Yes	Partial	Yes	Partial	Partial
Were the adverse events reported?	Yes	Yes	Yes	Yes	Yes	Yes	Yes	Yes	Yes	Yes
Were the conclusions of the study supported by results?	Yes	Yes	Yes	Yes	Yes	Yes	Yes	Yes	Yes	Yes
Were both competing interests and sources of support for the study reported?	Yes	Yes	Yes	Yes	Yes	Yes	Yes	Yes	Yes	Unclear
Global Judgement	High	High	High	High	High	High	High	High	High	High

Institute of health economics case series quality appraisal checklist [16]	Rai and Manav, 2021 [86]	Schiphouwer et al. 2017 [124]	Gao et al. 2020 [108]	Retzky et al. 2023 [58]	Gondolfo et al. 2022 [28]	Deasey et al. 2020 [84]	Huo et al. 2024 [112]	Chilelli et al. 2024 [25]
Was the hypothesis/aim/objective of the study clearly stated?	Yes	Yes	Yes	Yes	Yes	Yes	Yes	Yes
Was the study conducted prospectively?	No	No	No	No	No	No	No	No
Were the cases collected in more than one centre?	No	No	No	No	No	No	No	Yes
Were patients recruited consecutively?	Unclear	Unclear	Unclear	Unclear	Unclear	Unclear	Unclear	Unclear
Were the characteristics of the patients included in the study described?	Yes	Yes	Yes	Yes	Yes	Yes	Yes	Yes
Were the eligibility criteria (i.e., inclusion and exclusion criteria) for entry into the study clearly stated?	Yes	Yes	Yes	Yes	Yes	Yes	Yes	Yes
Did patients enter the study at a similar point in the disease?	Yes	Yes	Yes	Yes	Yes	Yes	Yes	No
Was the intervention of interest clearly described?	Yes	Yes	Yes	Yes	Yes	Yes	Yes	Yes
Were additional interventions (co-interventions) clearly described?	Yes	Yes	No	No	Yes	Yes	No	No
Were relevant outcome measures established a priori?	Unclear	Unclear	Unclear	Unclear	Unclear	Unclear	Unclear	Unclear
Were outcome assessors blinded to the intervention that patients received?	Unclear	Unclear	Unclear	Unclear	Unclear	Unclear	Unclear	Unclear
Were the relevant outcomes measured using appropriate objective/subjective methods?	Yes	Yes	Yes	Yes	Yes	Yes	Yes	Yes
Were the statistical tests used to assess the relevant outcomes appropriate?	Partial	Partial	Partial	Partial	Partial	Yes	Yes	Yes
Was follow-up long enough for important events and outcomes to occur?	No	No	Yes	Partial	Yes	Partial	Yes	Yes
Were losses to follow-up reported?	No	Yes	Yes	Yes	Yes	Yes	No	NA
Did the study provide estimates of random variability in the data analysis of relevant outcomes?	Partial	Yes	Yes	Yes	Yes	Yes	Yes	Yes
Were the adverse events reported?	Unclear	Yes	Yes	Yes	Yes	Yes	Yes	Yes
Were the conclusions of the study supported by results?	Yes	Yes	Yes	Yes	Yes	Yes	Yes	Yes
Were both competing interests and sources of support for the study reported?	Yes	Yes	Unclear	Unclear	Yes	Yes	Unclear	Yes
Global Judgement	High	High	High	High	High	High	High	High

Rob 2 tool for assessing risk of bias in randomised trials [19]	Astur et al. 2015 [25]	Ercan et al. 2021 [26]	Zhang et al. 2024 [50]
Domain 1: Risk of bias arising from the randomization process			
1.1 Was the allocation sequence random?	Yes	Yes	Yes
1.2 Was the allocation sequence concealed until participants were enrolled and assigned to interventions?	Probably Yes	Yes	Yes
1.3 Did baseline differences between intervention groups suggest a problem with the randomization process?	No	No	No
Risk-of-bias judgement	Low	Low	Low
What is the predicted direction of bias arising from the randomization process?	NA	NA	NA
Domain 2: Risk of bias due to deviations from the intended interventions (*effect of assignment to intervention*)			
2.1. Were participants aware of their assigned intervention during the trial?	Probably No	Probably No	Yes
2.2. Were carers and people delivering the interventions aware of participants’ assigned intervention during the trial?	Yes	Yes	Yes
2.3. If Y/PY/NI to 2.1 or 2.2: Were there deviations from the intended intervention that arose because of the trial context?	No	No	No
2.4 If Y/PY to 2.3: Were these deviations likely to have affected the outcome?	NA	NA	NA
2.5. If Y/PY/NI to 2.4: Were these deviations from intended intervention balanced between groups?	NA	NA	NA
2.6 Was an appropriate analysis used to estimate the effect of assignment to intervention?	Yes	Yes	Yes
2.7 If N/PN/NI to 2.6: Was there potential for a substantial impact (on the result) of the failure to analyse participants in the group to which they were randomized?	NA	NA	NA
Risk-of-bias judgement	Low	Low	Low
What is the predicted direction of bias due to deviations from intended interventions?	NA	NA	NA
Domain 3: Missing outcome data			
3.1 Were data for this outcome available for all, or nearly all, participants randomized?	Yes	Yes	Yes
3.2 If N/PN/NI to 3.1: Is there evidence that the result was No biased by missing outcome data?	NA	NA	NA
3.3 If N/PN to 3.2: Could missingness in the outcome depend on its true value?	NA	NA	NA
3.4 If Y/PY/NI to 3.3: Is it likely that missingness in the outcome depended on its true value?	NA	NA	NA
Risk-of-bias judgement	Low	Low	Low
What is the predicted direction of bias due to missing outcome data?	NA	NA	NA
Domain 4: Risk of bias in measurement of the outcome			
4.1 Was the method of measuring the outcome inappropriate?	Yes	Yes	No
4.2 Could measurement or ascertainment of the outcome have differed between intervention groups?	Unclear	No	No
4.3 If N/PN/NI to 4.1 and 4.2: Were outcome assessors aware of the intervention received by study participants?	Unclear	Unclear	No
4.4 If Y/PY/NI to 4.3: Could assessment of the outcome have been influenced by knowledge of intervention received?	Probably Yes	Probably No	NA
4.5 If Y/PY/NI to 4.4: Is it likely that assessment of the outcome was influenced by knowledge of intervention received?	Probably Yes	NA	NA
Risk-of-bias judgement	Some concerns	Low	Low
What is the predicted direction of bias in measurement of the outcome?	Unpredictable	NA	NA
Domain 5: Risk of bias in selection of the reported result			
5.1 Were the data that produced this result analyse in accordance with a pre-specified analysis plan that was finalized before unblinded outcome data were available for analysis?	Unclear	Unclear	Yes
Is the numerical result being assessed likely to have been selected, on the basis of the results, from…			
5.2. … multiple eligible outcome measurements (e.g., scales, definitions, time points) within the outcome domain?	Yes	Yes	No
5.3 … multiple eligible analyses of the data?	Yes	Yes	No
Risk-of-bias judgement	Some concerns	Some concerns	Low
Study risk of bias judgement	Some concerns	Some concerns	Low

Clarity tool for Cohort studies [17]	Kim et al. 2021 [32]	Klueh et al. 2024 [54]	Markes et al. 2024 [33]	Moran et al. 2025 [85]	Shankar et al. 2024 [40]	Rai et al. 2021 [86]	Hu et al. 2025 [111]
Was selection of exposed and Non-exposed cohorts drawn from the same population?	Probably Yes	Definitely Yes	Definitely Yes	Probably Yes	Definitely Yes	Definitely Yes	Definitely Yes
Can we be confident in the assessment of exposure?	Definitely Yes	Probably Yes	Definitely Yes	Definitely Yes	Definitely Yes	Definitely Yes	Definitely Yes
Can we be confident that the outcome of interest was No present at the start of the study?	Probably Yes	Probably Yes	Definitely Yes	Definitely Yes	Definitely Yes	Definitely Yes	Definitely Yes
Did the study match exposed and unexposed for all variables that are associated with the outcome of interest, or did the statistical analysis adjust for these prognostic values?	Probably No	Probably Yes	Probably Yes	Definitely No	Definitely Yes	Probably No	Definitely Yes
Can we be confident in the assessment of the presence of absence of prognostic factors?	Probably Yes	Probably Yes	Probably No	Probably Yes	Definitely Yes	Definitely Yes	Definitely Yes
Can we be confident in the assessment of outcome?	Probably Yes	Probably Yes	Probably Yes	Probably Yes	Definitely Yes	Definitely Yes	Probably No
Was the follow up of cohorts adequate?	Probably No	Probably No	Probably Yes	Probably Yes	Definitely Yes	Definitely Yes	Definitely Yes
Were co-interventions similar between groups?	Probably Yes	Probably No	Probably No	Probably Yes	Definitely Yes	Definitely Yes	Definitely Yes
Risk of bias assessment	High	Some concerns	High	High	Low	Some concerns	Some concerns

Clarity tool for case control studies [17]	Howells and Eldridge, 2012 [51]	Kita et al. 2015 [90]	Ye M et al. 2020 [80]
1. Can we be confident in the assessment of exposure?	Definitely Yes	Definitely Yes	Probably Yes
2. Can we be confident that cases had developed the outcome of interest and controls had No?	Definitely Yes	Probably Yes	Probably Yes
3. Were the cases (those who were exposed and developed the outcome of interest) properly selected?	Definitely Yes	Probably Yes	Probably No
4. Were the controls (those who were exposed and did No develop the outcome of interest) properly selected?	Definitely Yes	Definitely Yes	Probably Yes
5. Were cases and controls matched according to important prognostic variables or was statistical adjustment carried out for those variables?	Definitely Yes	Definitely Yes	Probably Yes
Risk of bias	Low	Some concerns	High

Rob 2 tool for assessing risk of bias in non-randomized trials of intervention [18]	Niu et al. 2018 [122]	Zheng et al. 2019 [81]
Domain 1: bias due to confounding		
1.1 Is there potential for confounding of the effect of intervention in this study?	Probably No	No
1.2 Was the analysis based on splitting participants follow up time according to intervention received?	No	No
1.3 Were intervention discontinuations or switches likely to be related to factors that are prognostic for the outcome?	No	No
1.4 Did the authors use an appropriate analysis method that controlled for all the important confounding domains?	Unclear	Probably Yes
1.5 Were confounding domains that were controlled for measured validly and reliably by the variables available in this study?	Unclear	Yes
1.6 Did the authors control for any post-intervention variables that could have been affected by the intervention?	Unclear	Unclear
1.7 Did the authors use an appropriate analysis method that adjusted for all the important confounding domains and for time varying confounding?	Unclear	Probably Yes
1.8 Were confounding domains that were adjusted for measured validly and reliably by the variables available in this study?	Unclear	Unclear
Risk of bias judgement	Some concerns	Some concerns
Domain 2: Bias in selection of participants into the study		
2.1. Was selection of participants into the study (or into the analysis) based on participant characteristics observed after the start of intervention?	No	No
2.2 Were the post-intervention variables that influenced selection likely to be associated with intervention?	No	No
2.3 Were the post-intervention variables that influenced selection likely to be influenced by the outcome or a cause of the outcome?	No	No
2.4 Do start of follow-up and start of intervention coincide for most participants?	Yes	Yes
2.5 Were adjustment techniques used that are likely to correct for the presence of selection biases?	No	No
Risk of bias judgement	Some concerns	Some concerns
Domain 3: Bias in classification of interventions		
3.1 Were intervention groups clearly defined?	Yes	Yes
3.2 Was the information used to define intervention groups recorded at the start of the intervention?	Unclear	Yes
3.3 Could classification of intervention status have been affected by knowledge of the outcome or risk of the outcome?	Probably Yes	Probably Yes
Risk of bias judgement	High	High
Domain 4: Bias due to deviations from intended interventions		
4.1. Were there deviations from the intended intervention beyond what would be expected in usual practice?	No	No
4.2 Were these deviations from intended intervention unbalanced between groups and likely to have affected the outcome?	No	No
Risk of bias judgement	Low	Low
Domain 5: Bias due to missing data		
5.1 Were outcome data available for all, or nearly all, participants?	Yes	Yes
5.2 Were participants excluded due to missing data on intervention status?	No	No
5.3 Were participants excluded due to missing data on other variables needed for the analysis?	No	No
5.4 Are the proportion of participants and reasons for missing data similar across interventions?	Yes	Yes
5.5 Is there evidence that results were robust to the presence of missing data?	Yes	Yes
Risk of bias judgement	Low	Low
Domain 6: Bias in measurement of outcomes		
6.1 Could the outcome measure have been influenced by knowledge of the intervention received?	Yes	Yes
6.2 Were outcome assessors aware of the intervention received by study participants?	No	Unclear
6.3 Were the methods of outcome assessment comparable across intervention groups?	Yes	Yes
6.4 Were any systematic errors in measurement of the outcome related to intervention received?	No	No
Risk of bias judgement	Some concerns	Some concerns
Domain 7: Bias in selection of the reported result		
Is the reported effect estimate likely to be selected, on the basis of the results, from 7.1. … multiple outcome measurements within the outcome domain?	No	No
7.2 … multiple analyses of the intervention-outcome relationship?	No	No
7.3 … different subgroups?	No	No
Risk of bias judgement	Low	Low
Study risk of bias judgement	High	High

## Appendix 4

See Table 6

**Table 6:** Number of fractures, graft types used, tunnels drilled, and concomitant procedures in included studies

Study	Number of patients	Number of fractures	Graft used	Tunnel drilled	Tunnel location	Anchor type	Concomitant procedures
Acharya et al. 2015 [46]	18	0	Semitendinosus tendon autograft	Yes	Both femoral and patellar tunnels	Interference screw	None
Allahabadi and Pandya, 2022 [101]	20	1	Gracilis tendon allograft	NR	NR	NR	None
Ambrožič and Novak, 2016 [72]	29	0	Quadriceps tendon autograft	Yes	Femoral tunnel only	Suture anchor	None
Arshi et al. 2016 [23]	6190	588	NR	NR	NR	NR	Tibial Tubercle Osteotomy, Microfracture Surgery, Chondroplasty, Osteochondral Grafting, Loose Cartilage Removal, Lateral Retinacular Release
Astur et al. 2015 [24]	58	1	Gracilis tendon autograft	Yes	Group 1: both femoral and patellar tunnels, Group 2: only femoral tunnel	Interference screw	None
Bachman et al. 2024 [52]	31	0	Hamstring graft	NR	NR	Transphyseal screws (20 knees)	17 knees: tension band plates
Chatterton et al. 2018 [83]	23	0	Semitendinosus autograft: 13 patients Allograft: 8 patients	Yes	Femoral tunnel only	NR	6 patients had concomitant TTO performed
Christiansen et al. 2008 [93]	44	1	Gracilis tendon autograft	Yes	Both femoral and patellar tunnels	Interference screw	Tibial tuberosity transfer
Deie et al. 2011 [102]	29	0	Semitendinosus tendon autograft	Yes	Femoral tunnel only	Femur – spike staple securing tendon/bone plug; Patella – soft-tissue suturing to periosteum (no hardware)	None
Deo et al. 2023 [103]	85	0	Synthetic graft (e.g., polyester, LARS)	Yes	Femoral tunnel only	Patella – suture anchors (2 × 2.4 mm); Femur – bio-absorbable interference screw	None
Devgan et al. 2019 [104]	20	0	Semitendinosus tendon autograft	Yes	Both femoral and patellar tunnels	Interference screw	Arthroscopic loose-body removal, chondral lesion debridement, meniscal debridement (when required)
Dong et al. 2022 [105]	45	0	Semitendinosus tendon autograft	Yes	Both femoral and patellar tunnels	Femoral fixation: 7 mm interference screw Patellar fixation: bone tunnel with sutures	VMO advancement
Enderlein et al. 2014 [106]	224	0	Gracilis tendon autograft	Yes	Both femoral and patellar tunnels	Interference screw	Patients with increased TTTG distance recieved tuberosity transfer, tibial tuberosity osteotomy in 23% of patients, chondral procedures where approrpriate.
Ercan et al. 2021 [26]	80	0	Semitendinosus tendon autograft	Yes	Patellar tunnel only	Bioabsorbable BioRCI interference screw	NR
Fadel and Hosni, 2020 [71]	34	0	Quadriceps tendon autograft	Yes	Femoral tunnel only	Patella – soft-tissue looping/no hardware Femur – bio-absorbable interference screw (7 × 30 mm)	Arthroscopic chondroplasty in 5 knees; no bony realignment
Fisher et al. 2021 [73]	34	0	Quadriceps tendon autograft	Yes	Femoral tunnel only	Femoral fixation: 7 mm bioabsorbable interference screw with sutures	Chondroplasty (*n* = 13)
Fitzgerald et al. 2023 [27]	29,539	141	NR	NR	NR	NR	NR
Fuji et al. 2021[107]	24	0	Semitendinosus tendon autograft	Yes	Both femoral and patellar tunnels	Femoral fixation : tenodesis screw (*n* = 6), Endobutton CL (*n* = 7) and ToggleLoc fixation device (*n* = 14), Patellar fixation: Endobutton	None
Gomes, 1992 [88]	30	1	Synthetic graft (e.g., polyester, LARS)	Yes	Patellar tunnel only	Femoral fixation: bone washer and screw/metallic washer, Patellar fixation: knot (lateral entrance of tunnel is enlarged to avoid excessive knot protuberance)	Chondral procedures (e.g. microfracture, chondroplasty) and lateral retinacular releas
Gorbaty et al. 2021 [29]	486	4	NR	NR	NR	NR	None
Goyal et al. 2018 [62]	23	0	Semitendinosus tendon autograft	Yes	Patellar tunnel only	Suture anchor	None
Han et al. 2010 [109]	52	0	Semitendinosus tendon autograft, gracilis autograft if ST insufficient	Yes	Both femoral and patellar tunnels	Femoral fixation: 7 × 25 mm (Depuy Mitek) interference screw, Patellar fixation: looped graft	lateral retinacular release and chondral procedures as necessary
Han et al. 2022 [30]	57	0	Semitendinosus tendon autograft	Yes	NR	26 cases received graft fixation with suture anchors 31 cases received graft fixation with semi-tunneling polyetheretherketone suture anchors	NR
Hiemstra et al. 2021 [110]	363	1	NR	NR	NR	NR	NR
Hohn and Pandya, 2017 [53]	25	1	Gracilis tendon autograft	Yes	Both femoral and patellar tunnels	Interference screw	Lateral retinacular release (*n* = 5), chondroplasty (*n* = 9), loose-body removal (*n* = 3), patellar microfracture (*n* = 1), partial meniscectomy (*n* = 2)
Hong et al. 2024 [77]	27	0	Semitendinosus tendon autograft	Yes	Femoral tunnel only	Patella – suture anchor (metal); Femur – bio-absorbable interference screw	Lateral retinacular release in 7 knees; chondroplasty in 5 knees
Hopper et al. 2014 [94]	68	4	Gracilis tendon autograft (48 knees), ST graft in 15 and combined gracilis + ST graft in 9	Yes	Both femoral and patellar tunnels	Patella – interference screw in bone tunnel; Femur – usually interference screw (61/72), occasionally suture anchor (11/72)	TTO performed in 22 knees
Howells et al. 2012 [89]	193	1	Semitendinosus tendon autograft	Yes	Both femoral and patellar tunnels	Patella – Endobutton (suspensory); Femur – Interference screw	Tibial tubercle distalisation (*n* = 5) and standard arthroscopic chondral procedures
Howells and Eldridge, 2012 [51]	75	1	Semitendinosus tendon autograft	Yes	Both femoral and patellar tunnels	Patella – Endobutton (suspensory); Femur – Interference screw	None
Kang et al. 2014 [31]	45	0	Semitendinosus tendon autograft	Yes	Femoral tunnel only	Femoral fixation: interference screw, Patellar fixation: sutured fixation to patellar soft tissue and retinaculum	None
Kim et al. 2021 [32]	81	1	MPFLr alone: 8 autograft and 28 allograft, MPFLr with TTO : 15 autograft and 30 allograft. Graft material apparently semitentinosus or tibialis anterior when allograft, unclear when autograft.	Yes	Femoral tunnel only	MPFLr alone - femoral fixation: interference screw (*n* = 8) and suspensory fixation (*n* = 28) and patellar fixation : suture anchor (*n* = 30) and tendon sling (*n* = 6). MPFLr with TTO - femoral fixation: interference screw (*n* = 15) and suspensory fixation (*n* = 30) and patellar fixation: suture anchor (*n* = 33) and tendon sling (*n* = 12)	MPFLr alone: lateral release (*n* = 6) and loose body removal (*n* = 2), MPFLr with TTO : lateral release (*n* = 8) and loose body removal (*n* = 4)
Kita et al. 2012 [91]	24	1	Semitendinosus tendon autograft	Yes	Both femoral and patellar tunnels	Endobutton or suspensory fixation	None
Kita et al. 2015 [90]	42	3	Semitendinosus tendon autograft	Yes	Femoral tunnel only	Endobutton or suspensory fixation	None
Kita et al. 2017 [113]	23	0	Semitendinosus tendon autograft	Yes	Both femoral and patellar tunnels	Endobutton or suspensory fixation	Lateral retinacular release
Klueh et al. 2024 [54]	48	0	Semitendinosus tendon allograft	Yes	Femoral tunnel only	soft tissue screw	NR
Kodkani et al. 2016 [67]	50	1	primarily gracilis tendon (minimum 210 mm length), with semitendinosus used in some cases (e.g. in smaller/non-athletic female patients or if gracilis was insufficient)	No	No bony tunnels (soft tissue fixation only)	Soft tissue looping (e.g., around adductor tubercle)	None
Kumahashi et al. 2012 [55]	5	0	Semitendinosus tendon autograft	Yes	Patellar tunnel only	Femoral fixation: soft tissue looping (MCL pulley) Patellar fixation: 5 × 9 mm interference screw and sutures	Lateral reticular release (*n* = 4) and VMO advancement as necessary
Kumahashi et al. 2014 [114]	17	0	Semitendinosus tendon autograft	Yes	Both femoral and patellar tunnels	Interference screw	Selective lateral retinacular release when indicated
Kumar et al. 2014 [9]	30	0	Gracilis tendon autograft	Yes	Both femoral and patellar tunnels	Patella – Endobutton (suspensory fixation); Femur – bioabsorbable interference screw (staple used only if cost constrained).	Microfracture for grade III–IV chondral lesions; debridement for grade I–II lesions.
Kumar et al. 2018 [56]	59	0	Allograft	Yes	Patellar tunnel only	Interference screw	Loose body removal
Lin et al. 2015 [115]	18	0	Semitendinosus tendon autograft	Yes	soft tissue + femoral	2 × 3.5 mm double-loaded metal suture anchor	NR
Liu et al. 2018 [79]	121	0	semitendinosus tendon autograft + semitendinosus allograft	Yes	Both femoral and patellar tunnels	Patella: bone tunnel with sutures (initial 5 mm socket) → later suture anchors; Femur: bio-absorbable interference screw	*Lateral retinacular release* (63%), *chondral procedures* (microfracture 9%, DeNovo 3%), *meniscal surgery* (4%); no TTO
Maione et al. 2022 [116]	46	0	Synthetic in some patients and autologous semitendinosus in others (choice determined on the basis of patient features by the surgeon)	Yes	Femoral tunnel only	Femoral fixation: 7 × 25 mm interference screw, Patellar fixation: suture anchors	None
Marigi et al. 2023 [117]	56	1	autografts (gracilis, semitendinosus) and allograft sources (gracilis, semitendinosus)	NR	NR	Suture anchor	MPFL repair or reconstruction, tibial tubercle osteotomy, lateral retinacular release, and medial retinacular reefing
Markes et al. 2024 [33]	70,070	79	NR	NR	NR	NR	NR
Matthews and Schranz, 2010 [118]	21	0	Gracilis or semitendinosus tendon autograft.	Yes	Both femoral and patellar tunnels	Patella – bone tunnel with sutures (no hardware); Femur – titanium interference screw. Early cases used femoral suturing, later all used screws.	None
Mikashima et al. 2006 [44]	24	2	Semitendinosus tendon autograft	NR	Two techniques: (A) no bony tunnels (sutured to periosteum/fibrous tissue); (B) patellar tunnel only (4.5 mm drill from medial margin to anterior surface). Femoral side: suspensory button; femoral tunnel not specified.	Femur: endobutton/suspensory fixation. Patella: bone tunnel with sutures or soft-tissue looping/periosteal sutures	None
Mochizuki et al. 2019 [82]	24	0	Gracilis tendon autograft	Yes	Femoral tunnel only	Suture anchor	Loose-body removal (*n* = 5), chondral procedures, loose-body removal; meniscal repair (*n* = 2)
Mohammed et al. 2017 [119]	53	0	Gracilis tendon was used in all patients except in one patient in which semitendinosus was used	Yes	Patellar tunnel only	Group S: Endobutton CL Fixation Device (Smith & Nephew) Group D: 2 × 4.75 mm BioComposite SwiveLock anchors (Arthrex)	29 knees (group S): single tunnel
Monllau et al. 2016 [120]	35	0	Gracilis tendon autograft	Yes	Patellar tunnel only	Bone tunnel with sutures (patella) and Soft-tissue looping around adductor magnus (femur)	29 knees (group D) two tunnels
Moran et al. 2025 [85]	560	1	Gracilis semitendinosus autograft or looped hamstring tendon graft	Yes	Patellar tunnel only	Suture anchor	TTO in 298 knees
Mulliez et al. 2017 [68]	124	1	Gracilis tendon autograft (117 cases); semitendinosus tendon autograft (12 knees).	No	No bony tunnels (soft tissue fixation only)	Suture anchor	Concomitant transposition of tibial tubercle in 38 knees
Nazeer et al. 2023 [69]	29	0	Semitendinosus tendon autograft	No	No bony tunnels (soft tissue fixation only)	Soft tissue looping (e.g., around adductor tubercle)	Patellofemoral chondroplasty
Neyret et al. 2012 [45]	32	0	Autograft; semitendinous [30] or gracilis tendon [5]	NR	NR	NR	Tibial tuberosity transfer
Niu et al. 2018 [122]	64	0	Semitendinosus tendon autograft	NR	NR	No. 2 FiberWire sutures	Lateral retinacular release
Niu et al. 2017 [121]	30	0	Semitendinosus tendon autograft	Yes	Both femoral and patellar tunnels	Femoral fixation: 6 × 10 mmbioabsorbable interference screw, Patellar fixation: FiberWire suture fixation over lateral bone bridge	Tibial tubercle transfer and lateral retinacular release
Ntagiopoulos et al. 2024 [34]	24	0	Semitendinosus tendon autograft	Yes	Both femoral and patellar tunnels	Bone tunnel with sutures	Meniscal repair:
Özel et al. 2025 [123]	51	0	Semitendinosus tendon autograft (ST) and quadriceps tendon autograft (QT, pedicled)	Yes	ST group: both femoral & patellar tunnels (two patellar tunnels; femoral tunnel at Schöttle point). QT group: femoral tunnel only; no patellar bony tunnels (pedicled graft fixed to patella with sutures)	Femoral: interference screw (both groups). Patella: bone tunnel with sutures (ST) vs. soft-tissue/periosteal sutures (soft-tissue looping) (QT)	Plica excision
Palacios et al. 2017 [35]	19	1	Semitendinosus tendon autograft	NR	NR	NR	ACL reconstruction
Panni et al. 2011 [92]	48	1	Semitendinosus tendon autograft	Yes	Both femoral and patellar tunnels	Interference screw + Bone tunnel with sutures	Meniscal debridement/meniscectomy when required
Parikh et al. 2013 [95]	154	6	Gracilis tendon autograft	Yes	Both femoral and patellar tunnels	Patella – bone tunnel with sutures; Femur – bio-absorbable interference screw	None routinely; lateral retinacular release performed only for habitual dislocations (rare)
Park et al. 2025 [36]	73	0	Allograft	No	NR	Tenodesis anchor at the distal adductor tubercle on the femur	TTO
Perkins et al. 2021 [37]	82	1	NR	NR	NR	NR	None
Reddy et al. 2022 [57]	57	2	Allograft	Yes	Patellar tunnel only	Interference screw	Trochleoplasty (*n* = 9), medial displacement tibial tubercle osteotomy (*n* = 3), distalization of the tibial tubercle for patella alta (*n* = 3)
Rhatomy et al. 2019 [38]	8	0	Quadriceps tendon autograft	Yes	Femoral tunnel only	resorbable interference screw (ConMed)	NR
Ronga et al. 2009 [63]	28	0	Gracilis tendon autograft (first choice) — semitendinosus autograft used in 5 patients when gracilis insufficient.	Yes	Both femoral and patellar tunnels	Patella: bone tunnels with looped graft (no hardware). Femur: bio-absorbable interference screw.	Arthroscopic micro-fracture or loose-body removal when osteochondral fragments were present
Sanguanjit et al. 2023 [39]	43	0	Quadriceps graft: 21 patients Hamstring graft: 22 pt	Yes	QTgraft: single femoral tunnel Hamstring graft: single patellar tunnel	Both: interference screw 6 × 25 mm	None
Shankar et al. 2024 [40]	10	0	gracilis or semitendinosus allografts	NR	NR	NR	Concomitant osteochondral allograft, concomitant TTO
Sherman et al. 2019 [47]	87	1	NR	NR	NR	NR	TTO in 32 patients (38 knees)
Sherman et al. 2020 [49]	25	1	Allograft	NR	NR	NR	NR
Sim et al. 2018 [125]	12	0	Semitendinosus tendon autograft	Yes	Femoral tunnel only	Bone tunnel with sutures	None
Song et al. 2014 [78]	20	0	Semitendinosus tendon autograft (gracilis autograft used in 2 cases)	Yes	Femoral tunnel only	Patella – suture anchors (metal Corkscrew, 5 mm); Femur – bio-absorbable interference screw	Lateral retinacular release (3 pts); chondroplasty (5 pts)
Song et al. 2019 [64]	13	0	Semitendinosus tendon autograft	Yes	Femoral tunnel only	Interference screw	‘4-in-one’ procedure – including tibial tubercle proximalization, extensive lateral release, tibial tubercle medialization, and medial patellofemoral ligament reconstruction]
Steiner et al. 2006 [75]	34	0	Adductor magnus tendon autograft (23), Quadriceps tendon autograft (6), Patellar tendon allograft (5)	Yes	Both femoral and patellar tunnels	Femoral side: 4 mm cancellous interference/lag screw into bone block; Patellar side: Bone tunnel with sutures/soft-tissue looping over patella	None
Sundararajan et al. 2015 [41]	22	0	Semitendinosus tendon autograft	Yes	Patellar tunnel only	Interference screw	None
Tang et al. 2015 [126]	16	0	Allograft	Yes	Femoral tunnel only	Interference screw	None
Teküstün et al. 2025 [76]	8	0	Semitendinosus tendon autograft (9 knees), quadriceps tendon autograft (2 knees)	Yes	Femoral tunnel only	Interference screw	Lateral retinacular release (*n* = 11), advancement of medial retinaculum (*n* = 11), TTO (*n* = 10), distal femoral closing osteotomy (*n* = 10), high tibial osteotomy (*n* = 1), trochleoplasty (*n* = 1)
Torkaman et al. 2015 [42]	15	0	Semitendinosus tendon autograft	Yes	Femoral tunnel only	Femoral fixation: bioabsorbable interference screw Patellar fixation: 2 x suture anchors in bony groove	None
Toritsuka et al. 2011 [127]	20	0	Semitendinosus tendon autograft	Yes	Both femoral and patellar tunnels	Endobutton or suspensory fixation	Lateral retinacular release (in first-time dislocations), open reduction & internal fixation of osteochondral fracture (ORIF), free-body removal
Valkering et al. 2017 [43]	31	0	Gracilis tendon autograft	Yes	NR	NR	None
van Gennip et al. 2014 [65]	9	0	Pedicled quadriceps tendon (*n* = 6), Semitendinosus tendon allograft (*n* = 2), Tibialis posterior tendon allograft (*n* = 1). Allografts used if patient tendon determined to be of poor quality.	Yes	Femoral tunnel only	Quads tendon autografts- Femoral fixation: soft tissue screw/anchor. Semitendinosus/tib posterior allografts- Femoral fixation: soft tissue screw/anchor, Patellar fixation: suture anchor	Lateral retinacular release in all, tuberosity transfer *n* = 2
von Engelhardt et al. 2017 [87]	30	0	Gracilis tendon autograft	Yes	Both femoral and patellar tunnels	Soft tissue looping (e.g., around adductor tubercle)	Trochleoplasty
Wang et al. 2016 [128]	26	0	Gracilis tendon autograft	Yes	Femoral tunnel only	Patella – suture anchor (plus bone groove & periosteal sutures); Femur – bioabsorbable interference screw	Arthroscopic lateral retinacular release when indicated; cartilage repair/debridement, meniscectomy/repair, loose-body removal as needed
Wu et al. 2025 [129]	68	0	Semitendinosus tendon autograft	Yes	Femoral tunnel only	Patella – suture anchor (metal); Femur – bio-absorbable interference screw	None
Xing et al. 2022 [130]	31	0	Semitendinosus tendon autograft	Yes	Patellar tunnel only [2]	Suture anchor	None
Yang et al. 2017 [70]	12	0	Peroneus longus tendon autograft	Yes	Femoral tunnel only	Femoral fixation: interference screw, Patellar fixation: suture anchors (2 × 5 mm spaced 1 cm apart)	Lateral retinacular release
Ye et al. 2020 [80]	65	0	Gracilis tendon autograft	Yes	Both femoral and patellar tunnels	Patella: bone tunnel with sutures; Femur: bio-absorbable interference screw	Lateral retinacular release performed in 17 knees (8 anchor, 9 transosseous)
Yoon et al. 2020 [132]	46	2	Tibialis allograft	Yes	Both femoral and patellar tunnels	Ttransosseous tunnel (*n* = 21) and suture anchor (*n* = 25)	Chondral procedures (e.g. microfracture, chondroplasty)
Yoon et al. 2024 [131]	55	1	Double-stranded tibialis anterior allograft	Yes	Femoral tunnel only	Suture anchor	None
Yoong et al. 2019 [48]	43	0	Gracilis tendon autograft	Yes	Patellar tunnel only	NR	NR
Zein et al. 2024 [60]	24	0	Quadriceps tendon autograft	No	No bony tunnels (soft tissue fixation only)	Soft tissue looping (e.g., around adductor tubercle)	Performed in 9/24 knees (37.5%): lateral release (*n* = 1), lateral retinacular lengthening (*n* = 2), patellar chondroplasty (*n* = 2), loose-body removal (*n* = 2), medial meniscus repair (*n* = 2).
Zein et al. 2024 [59]	11	0	Quadriceps tendon autograft	No	No bony tunnels (soft tissue fixation only)	NR	loose body removal (*n* = 1), meniscal repair (*n* = 2), V-Y quadriceps lengthening (*n* = 1), and patellar chondroplasty (*n* = 1)
Zhang et al. 2010 [133]	20	0	Allograft	Yes	Femoral tunnel only	Interference screw	None
Zhang et al. 2023 [61]	75	0	Semitendinosus tendon autograft	Yes	Both femoral and patellar tunnels	NR	For patients with increased femoral anteversion(> 30°) and external tibial torsion (> 30°), a double-levelderotational osteotomy (D‐DFO + D‐HTO) was some-times performed
Zhang et al. 2023 [134]	21	0	Peroneus longus tendon autograft	Yes	Patellar tunnel only	Bone tunnel with sutures	None
Zhang et al. 2024 [50]	50	0	Semitendinosus tendon autograft	Yes	Both femoral and patellar tunnels	Interference screw	TTO in all, lateral retinacular release performed as necessary
Zheng et al. 2018 [81]	30	0	Allograft	Yes	Femoral tunnel only	Patella – suture anchor (3 mm); Femur – bio-absorbable interference screw	Lateral retinacular release
Zhou et al. 2014 [66]	32	0	Semitendinosus tendon autograft	Yes	Both femoral and patellar tunnels	Femoral fixation: 7 × 10 mm bioabsorbable interference screw, Patellar fixation: bone tunnel with sutures	Lateral retinacular release as necessary
Zhou et al. 2023 [135]	64	0	Semitendinosus tendon autograft	Yes	Both femoral and patellar tunnels	NR	Derotational distal femur osteotomy
Zhu et al. 2017 [136]	25	0	Semitendinosus tendon autograft	Yes	Femoral tunnel only	Both suture anchor (two 3.5 mm double-loaded anchors) and interference screw (one absorbable screw to secure the graft in the fem tunnel)	Performed as needed: lateral retinacular release, chondral fragment fixatioNRemoval, meniscal repair or meniscectomy (exact numbers not reported)
Runer at al, 2024 [74]	64	0	Gracilis tendon autograft (*n* = 32), Quadriceps tendon autograft (*n* = 32)	Yes	GT: both femoral and patellar tunnels, QT: only femoral tunnel	GT- Femoral fixation: 6–8 × 28 mm interference screw Patellar fixation: 3.5 mm SwiveLock anchors. QT- Femoral fixation: 6–8 × 28 mm interference screw Patellar fixation:2 x resorbable sutures at medial patellar border	None
Rai et al. 2021 [86]	40	0	Not reported	Yes	Only femoral tunnel explicitly mentioned, patellar fixation not mentioned	Not reported	Tibial tubercle osteotomy
Schiphouwer et al. 2016 [124]	179	6	Hamstring autograft (preferably gracilis but number not specified)	Yes	Patellar tunnel only	Femoral fixation: loop around adductor tubercle tendon, Patellar fixation: absorbable sutures	MPFLr only *n* = 35 knees, MPFLr + TTO *n* = 134 knees, MPFLr + TTO + trochleoplasty *n* = 2 knees, MPFLr + trochleoplasty *n* = 21 knees
Chilelli et al. 2024 [25]	3152	12	Not reported	NR	Not reported	Not reported	Not reported
Gao et al. 2020 [108]	66	0	Gracilis tendon autograft	Yes	Both femoral and patellar tunnels	Femoral fixation: bioabsorbable interference screw, Patellar fixation: graft loop through bone tunnels	None
Retzky et al. 2023 [58]	56	0	Semitendinosus tendon autograft	Yes	Skeletally immature patients: femoral epiphysis, skeletally mature patients: distal femur. Both groups patellar tunnels.	Femoral fixation: bioabsorbable interference screw, Patellar fixation: suture anchors	None
Gondolfo et al. 2022 [28]	51	0	Hamstring autograft (Gracilis *n* = 11, semitendinosus *n* = 40)	Yes	Femoral tunnel all, patellar tunnel *n* = 4	Femoral fixation: interference screw, Patellar fixation: suture anchors except for *n* = 4 graft tunnel	None
Deasey et al. 2020 [84]	352	1	Semitendinosus tendon autograft	Yes	Patellar tunnel only (*n* = 215)	short, oblique tunnels were used for patellar fixation in 215 cases, and suture anchors were utilized in 169 cases	Tibial tubercle osteotomy and/or distalization of the tibial tubercle
Hu et al. 2025 [111]	112	1	Semitendinosus tendon autograft	Yes	Both femoral and patellar tunnels	Interference screw	None
Huo et al. 2024 [112]	130	0	Semitendinosus tendon autograft	Yes	Both femoral and patellar tunnels	A: No. 1 Ethicon nonabsorbable suture. Two No. 2 Fiberwire non-absorbable sutures were used to pull the free ends into the patellar tunnels and tied together for fixation over the lateral bone bridge. B + C: No. 2 Fiberwire nonabsorbable sutures in a whip-stitched style.	None
